# Multiscale modelling of biomolecular corona formation on metallic surfaces

**DOI:** 10.3762/bjnano.15.21

**Published:** 2024-02-13

**Authors:** Parinaz Mosaddeghi Amini, Ian Rouse, Julia Subbotina, Vladimir Lobaskin

**Affiliations:** 1 School of Physics, University College Dublin, Belfield, Dublin 4, Ireland

**Keywords:** all atomistic, aluminum, bionano interface, coarse grained model, lactose, milk protein, multiscale modelling, protein corona

## Abstract

In the realm of food industry, the choice of non-consumable materials used plays a crucial role in ensuring consumer safety and product quality. Aluminum is widely used in food packaging and food processing applications, including dairy products. However, the interaction between aluminum and milk content requires further investigation to understand its implications. In this work, we present the results of multiscale modelling of the interaction between various surfaces, that is (100), (110), and (111), of fcc aluminum with the most abundant milk proteins and lactose. Our approach combines atomistic molecular dynamics, a coarse-grained model of protein adsorption, and kinetic Monte Carlo simulations to predict the protein corona composition in the deposited milk layer on aluminum surfaces. We consider a simplified model of milk, which is composed of the six most abundant milk proteins found in natural cow milk and lactose, which is the most abundant sugar found in dairy. Through our study, we ranked selected proteins and lactose adsorption affinities based on their corresponding interaction strength with aluminum surfaces and predicted the content of the naturally forming biomolecular corona. Our comprehensive investigation sheds light on the implications of aluminum in food processing and packaging, particularly concerning its interaction with the most abundant milk proteins and lactose. By employing a multiscale modelling approach, we simulated the interaction between metallic aluminum surfaces and the proteins and lactose, considering different crystallographic orientations. The results of our study provide valuable insights into the mechanisms of lactose and protein deposition on aluminum surfaces, which can aid in the general understanding of protein corona formation.

## Introduction

**Figure 1:**
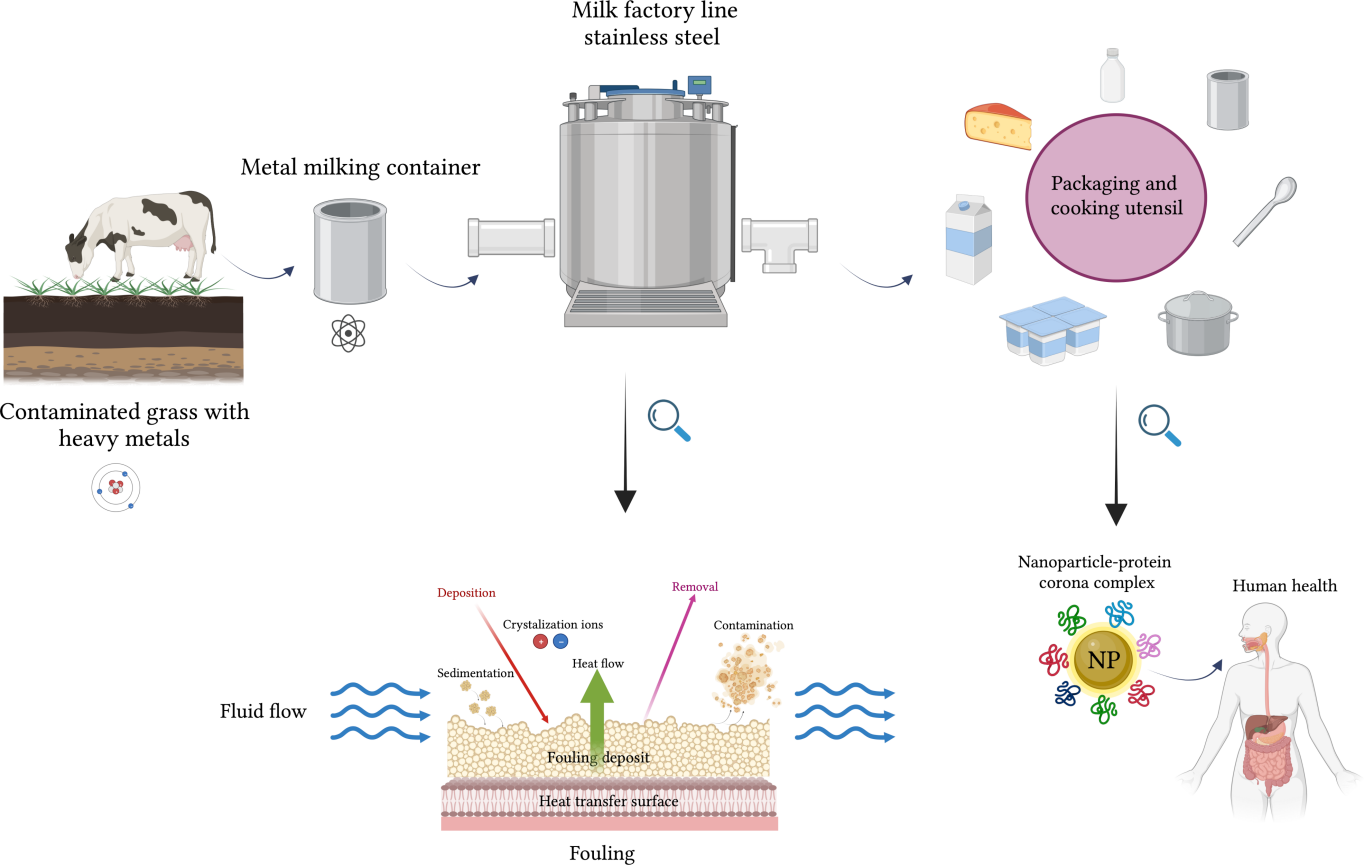
Schematic representation of the life cycle of dairy products, showcasing potential sources and pathways of contamination. It features the stages of grazing, collection, processing, and packaging. The relevant processes include surface fouling and milk contamination during food transformation as well as the formation of a protein corona on surfaces and nanoparticles after packaging. The figure was created with BioRender.com, https://biorender.com/. This content is not subject to CC BY 4.0.

The interface between biological systems and engineered materials has gained significant attention in recent years because of its wide range of applications, spanning from food to medicine and environmental science [1–2]. This interface plays a crucial role in ensuring the safety and quality of processed and packaged products. The selection of packaging materials and their interaction with biological components have emerged as critical determinants impacting the preservation, shelf life, and overall acceptability of dairy products [3]. Consequently, the interface between biologically relevant molecules and nanoscale materials, such as aluminum, has become an increasingly important and intriguing area of research [4]. For long-term storage and preservation of prepared food, the choice of containers and utensils made from specific materials is essential [5]. For example, it was shown that ripened cheese and cheese spreads acquire a higher aluminum content as compared to other milk products [6]. Aside from wrapping and container packaging, aluminum has found a wide popularity in other applications, such as manufacturing of kitchen utensils, cosmetics, and components for medical and scientific equipment [7]. Figure 1 presents a schematic contamination cycle of dairy products, showcasing potential sources and pathways of aluminum pollution. It illustrates the journey of milk from a cow grazing on grass contaminated with heavy metals, highlighting the crucial role of metallic containers, metal-based equipment, and kitchen utensils in maintaining product integrity. The figure further demonstrates the potential to introduce heavy metal contamination, including iron and aluminum, during processing and emphasizes the formation of a milk layer in form of a protein/lactose corona at the outer surface of macroscropic and micro- and nano-sized particulate after packaging. It also highlights the dynamic interactions at the bionano interface associated with potential human health hazards. Through biomolecule adsorption, change of conformation, and surface chemistry, foreign materials engage in a complex interplay of dynamic physicochemical interactions, kinetics, and thermodynamic exchanges that can lead to undesirable outcomes [1,8–10].

**Figure 2:**
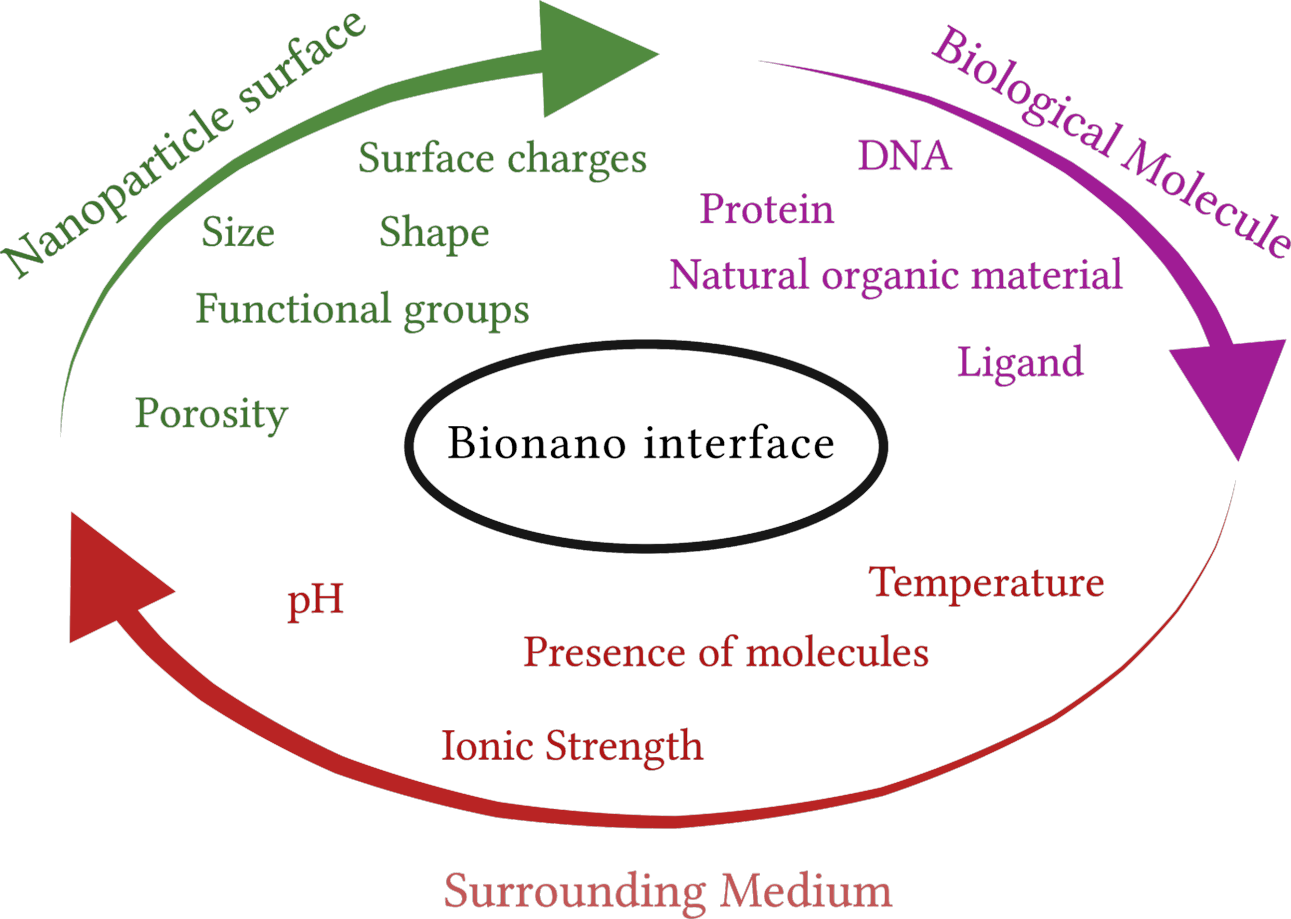
A chart of the main factors determining the structure of the bionano interface. The quantitative model comprises three essential aspects, that is, surface properties of the nanomaterial, the characteristics of the surrounding medium, and the biological factors at play.

In a more general context, the importance in understanding the mechanism of bionano interactions arises from the increasing awareness and concerns regarding the safety of nanoparticles (NPs) in relation to human and animal health. The toxicity of NPs is closely linked to their chemical aggressiveness and varies with their physicochemical properties, including surface area, charge, and reactivity. Understanding the intricate interplay between these properties and the biological systems is vital for assessing and mitigating any potential adverse effects associated with exposure to NPs [11]. To advance in this field, it is crucial to comprehend the underlying forces and molecular constituents that govern the interactions between biomolecules and metals. However, traditional safety assessment methods can be costly, time-consuming, and often involve animal studies. In this regard, in silico modelling offers a promising alternative that can predict the interactions of NPs with living organisms. By leveraging computational approaches, in silico modelling provides a humane and cost-effective means of obtaining the necessary information, thus aiding in the evaluation of NP safety and reducing reliance on animal experimentation [12–14]. Data-driven methods that rely on statistical analysis are employed for this purpose, particularly when sufficient data are available. These methods leverage the power of large datasets to identify patterns, trends, and correlations between metal properties and their interactions with biomolecules [15–18]. In recent years, researchers have focused on using physics-based models to understand the mechanisms underlying the formation of NP protein corona, a complex layer of biomolecules that surrounds NPs upon their exposure to biological fluids [19–20]. It is widely recognized that composition and configuration of the protein corona play a crucial role in determining the biochemical reactivity, sensitivity of NPs, as well as their cellular uptake and systemic transfer [21]. However, in order to develop predictive models, a deeper understanding of the interactions at the bionano interface and their relationship to material and protein properties is necessary. Gathering more information on these intricate interactions will facilitate the development of accurate predictive models, thereby advancing our ability to assess the behavior and potential implications of NPs in biological systems. The bionano interface can be broken down into three interconnected components: (i) the surface of the NP, which is influenced by its physicochemical composition, (ii) the interface between the solid NP and the surrounding liquid environment, where notable changes occur upon interaction, and (iii) the contact zone between the solid–liquid interface and biological substrates (Figure 2) [22].

**Figure 3:**
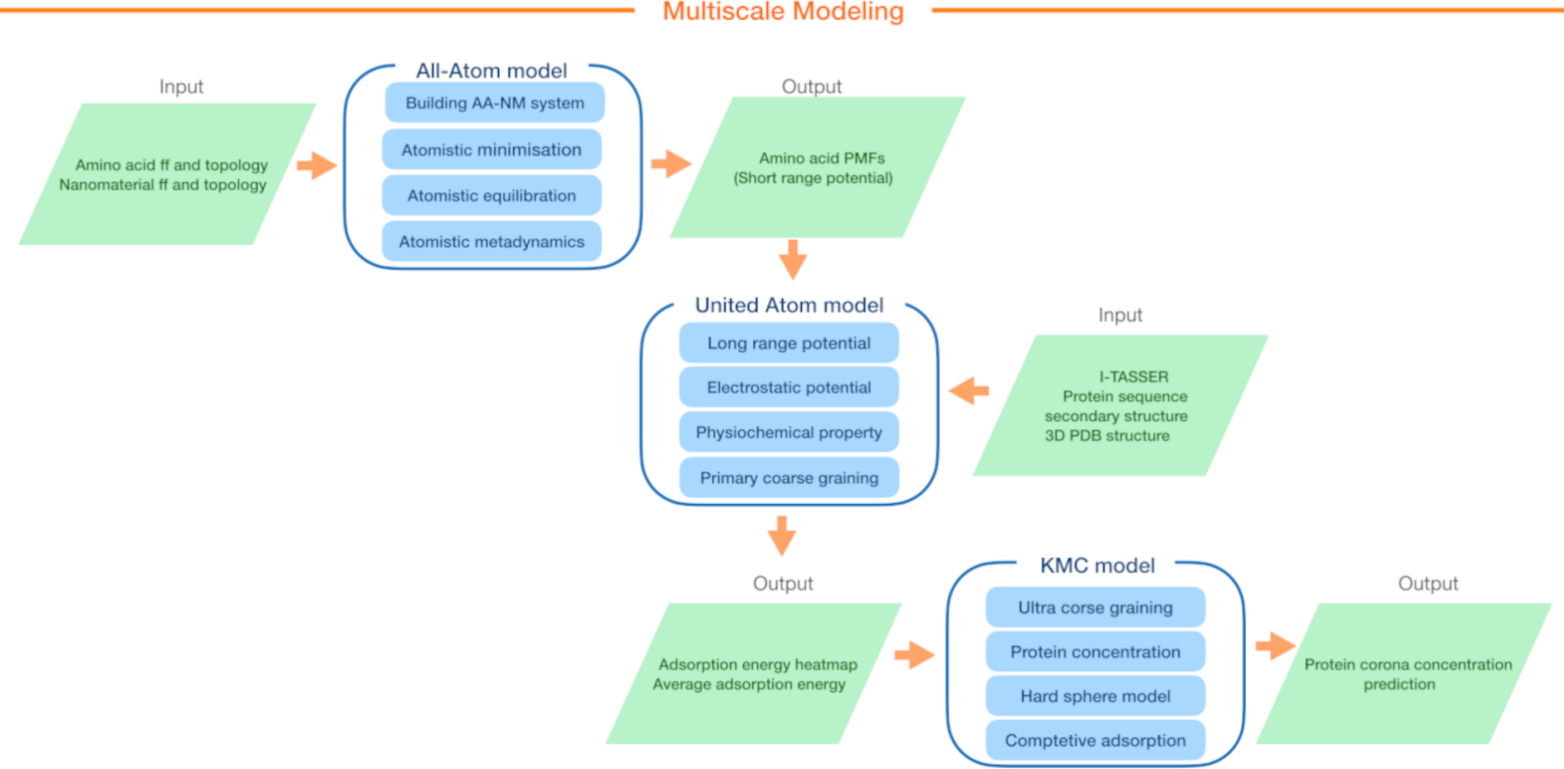
A dataflow scheme of the multiscale modelling approach implemented in this study, including an all-atom model of surface and AAs, a CG UnitedAtom model for the entire protein-surface interaction, and a CG competitive adsorption model. The figure provides an overview of input and output data at each scale.

In this work, we study bionano interactions involving metallic aluminum and common dairy biomolecules, namely lactose and the six most abundant milk proteins [23]. The main objective of our analysis is to computationally quantify the relative binding of these proteins on zero-valent aluminum surfaces based on their energy of adsorption and orientation. We employ a three-level multiscale method (as shown in Figure 3) to calculate the energies of adsorption and the content of the corona for these proteins on the selected surfaces. In the section “Results and Discussion”, we provide a detailed explanation of the theoretical model developed to study the interaction between protein and lactose with metals, as well as the rationale behind the parameterization scheme used. Subsequently, we discuss the simulation results and analyze the individual adsorption affinities predicted for molecules representing the biological aspect of the interface, including amino acids (AAs), milk proteins, and carbohydrates. Additionally, we examine the preferred orientations of these molecules upon adsorption and investigate the kinetics of competitive adsorption among the proteins and lactose, aiming to understand the process of protein deposition on metallic surfaces. Finally, the key insights gained from this study are summarized, highlighting the implications and potential applications of the findings.

## Results and Discussion

Here, we aim to predict the content of a biomolecular corona on a metallic aluminum surface. At the largest scale, our methodology employs a coarse-grained (CG) kinetic Monte Carlo (KMC) method [16] to simulate competitive adsorption of biomolecules onto the aluminum surface. To achieve this, we evaluate individual binding energies at various orientations (represented by heatmaps) for each selected protein immobilized on different fcc planes of the aluminum surface. These heatmaps for individual proteins are acquired through UnitedAtom (UA) simulations [24–25]. While the UA method has been parameterized for a range of rigid surfaces, including metals (Ag, Au, Cu, and Fe), oxides (TiO_2_, SiO_2_, and Fe_2_O_3_), carbonaceous NPs (graphene, carbon nanotubes, and carbon black), semiconductors (CdSe) [26], and polymers [27], it lacks the set of short-range potentials required for calculating milk protein-aluminum adsorption energies. Here, we compute potentials of mean force (PMF) for Al surfaces derived from explicit all-atom molecular dynamics simulations utilizing a previously established scheme [2,24,28]. These PMFs provide the input required to determine the adsorption energies between milk proteins and aluminum surfaces by using multiscale UA CG model, spanning from the atomistic level of description to the complete mesoscale model of the corona. Figure 3 shows the parameterization and simulation workflow, outlining different stages and components involved in the study.

### All-atoms short-range interaction modelling results

**Figure 4:**
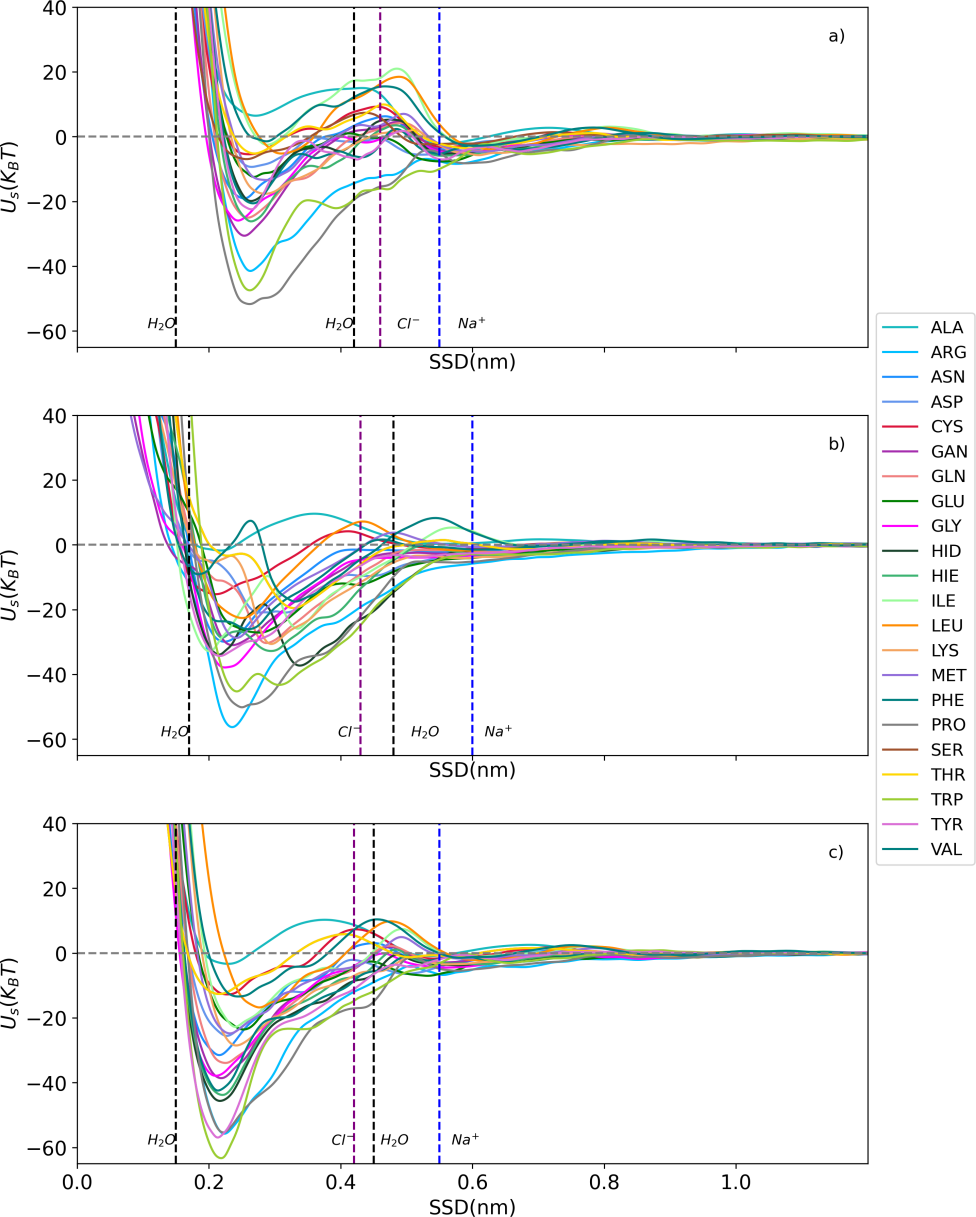
Adsorption free energy profiles of SCAs on three aluminum fcc slabs as a function of the surface separation distance (SSD). These profiles were calculated using all-atom AWT-MetaD. The vertical lines indicate the positions of water and ion layers. (a) Al(100), (b) Al(110), and (c) Al(111).

All-atom metadynamics simulations were conducted using GROMACS-2018.6 and PLUMED (PLUMED2-2.5.1.conda.5) software packages [29–31]. CHARMM-GUI/Nanomaterial Modeler was employed to construct the topology and force fields of three fcc surfaces of Al: (100), (110), and (111) [32]. The General Amber Force Field (GAFF) was utilized to model side-chains analogues (SCA) within the system [33–34]. The AMBER force field is a widely recognized and extensively validated force field that provides accurate descriptions of molecular systems [35]. We evaluated the short-range PMFs between 22 SCAs and an Al slab in a solvent environment comprising water and salt ions. The system’s pH value was maintained at a neutral level, and the NaCl salt concentration was set to 150 mM, mimicking the overall ionic strength of milk and equivalent to one salt molecule per 10 nm^3^. The system underwent equilibration for 1.0 ns under constant pressure conditions at 1.0 bar and a temperature of 300 K, following the NPT ensemble, employing Berendsen weak coupling method [36]. Subsequently, a pre-equilibration phase was conducted for 10 nanoseconds within the NVT ensemble. For the short-range interactions, the cut-off distance was defined as 1.0 nm. In the adaptive well-tempered metadynamics (AWT-MetaD) simulations, the adsorption energy was calculated at a temperature of 300 K, a pressure of 1.0 bar, and a neutral pH within the NVT ensemble. Additionally, we measured the interaction energy as a function of surface separation distance (SSD) as a collective variable, enabling a comprehensive analysis of the AA–NP interactions. For a detailed explanation of the method used in this study, please refer to previous reports [2,24,28] where the method has been described in depth. Figure 4 and dataset [37] show the obtained free energy of adsorption in units of *k*_B_*T*.

**Figure 5:**
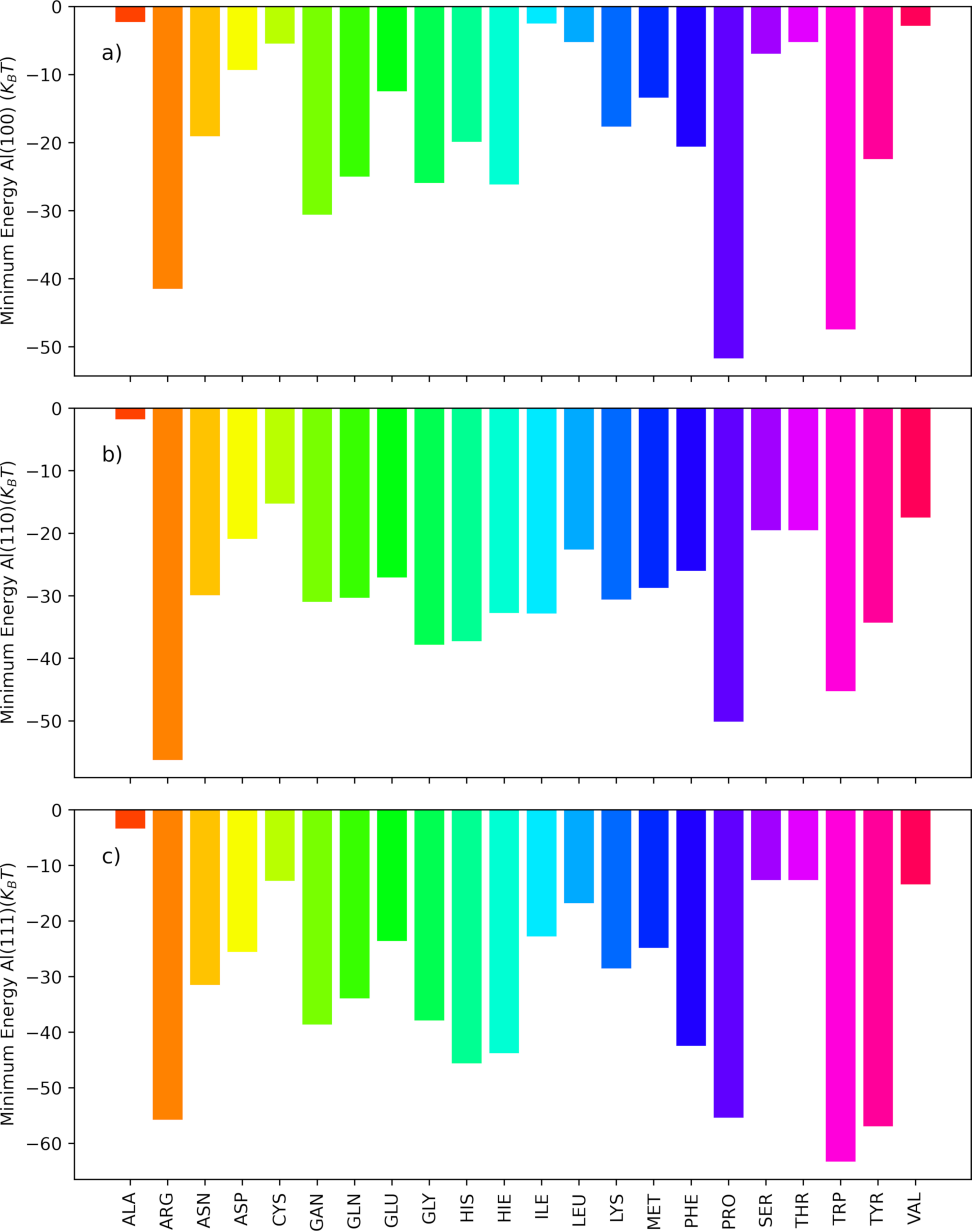
Minimum energy of adsorption (*k*_B_*T*) for each SCA on three Al fcc slabs obtained through all-atom simulations: (a) Al(100), (b) Al(110), and (c) Al(111). Notably, Al(111) exhibits a stronger binding affinity than Al(100) and Al(110).

The water density profiles obtained from MD simulations for the slab–water system in the context of Al surfaces revealed characteristics that were previously observed for other simulated metallic surfaces [2,28]. The profiles exhibited two distinct regions with elevated water density located approximately 0.15–0.18 nm and 0.42–0.48 nm away from the aluminum surface. These regions corresponded to the first and second water layers adjacent to the metal surface, respectively (as depicted in Supporting Information File 1, Figure S1). Further examination of the ion density profiles indicated the presence of sodium ions within a range of 0.55–0.60 nm and chloride ions within a range of 0.42–0.46 nm from the Al surface. Notably, the positions of the chloride ions align closely with the second water layer, while sodium ions are located past this layer, as marked by the blue and purple vertical dashed lines in Figure 4. This alignment suggests that the chloride ions integrate into the network of water molecules comprising the second adlayer. Additionally, the analysis of the PMFs revealed a significant minimum at a distance of 0.21–0.25 nm. Figure 5 shows the minimum energy values obtained for each AA on different facets of the aluminum surface (100, 110, and 111) in a bar chart.

**Figure 6:**
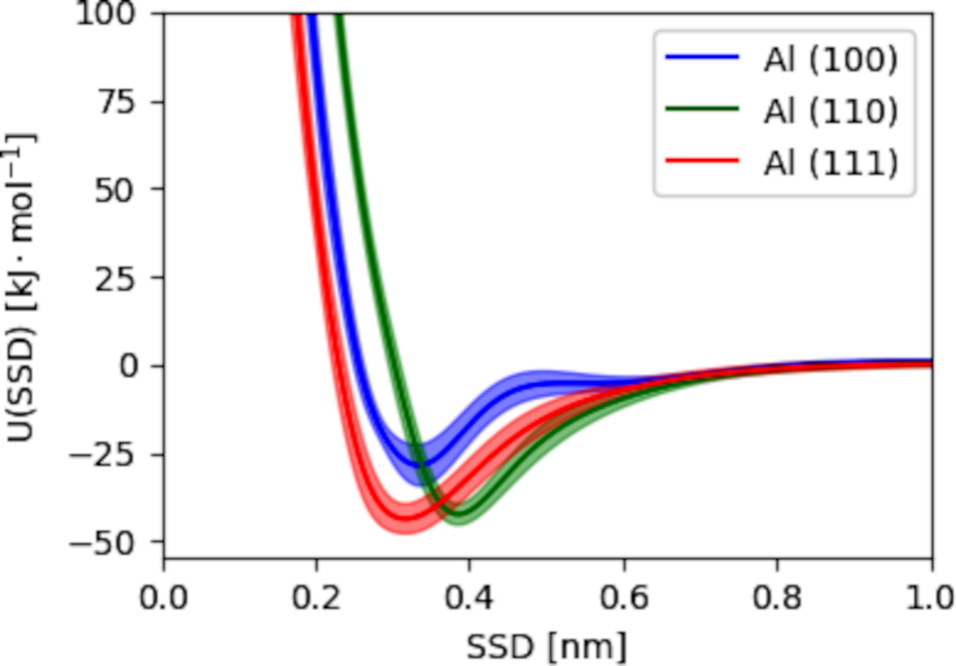
The interaction potential of glucose with the three Al surfaces predicted using the PMFPredictor Toolkit. The solid lines give the ensemble average of ten versions of the model while the shaded regions indicate the 95% confidence intervals.

A comparison of the adsorption energies on aluminum and iron surfaces reveals distinct preferences for different AAs. On aluminum surfaces, ARG, PRO, TRP, TYR AAs show the strongest attraction (−63.32*k*_B_*T* to −41.46*k*_B_*T*), followed by HIE, GLN, PHE, GAN (−43.86*k*_B_*T* to −20.85*k*_B_*T*). VAL, THR, SER, CYS, ALA exhibit the weakest attraction (−19.51*k*_B_*T* to −1.76*k*_B_*T*). On iron surfaces, charged and aromatic PRO, TYR, ARG, HIS AAs are strongly adsorbed (−91.29*k*_B_*T* to −43.34*k*_B_*T*), while hydrophobic VAL, LEU, ALA AAs show a weaker adhesion (−21.70*k*_B_*T* to 2.86*k*_B_*T*) [2]. We also show the PMF for glucose with aluminum surfaces, used as the basis for a model of lactose, a sugar highly present in milk, as discussed later, computed using the PMFPredictor software in Figure 6 [38].

### Protein–NP interactions

To further understand the adsorption energy and orientation of each individual protein, a primary coarse-graining step was performed. In this part, we use the UA model to predict the protein–NP binding energies. This model takes into account various factors, such as the material’s chemical composition, size, shape, surface roughness, charge, functionalization, and hydrophobicity, when constructing CG models for the bionano interface. The UA model simplifies the protein–NP interactions by representing proteins as rigid structures composed of 20 AA types, each represented by a single bead. This interaction is described through a short-range surface non-bonded potential (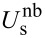) (including van der Waals (vdW) repulsion and solvent effects), a long-range core vdW potential (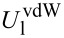), and an electrostatic potential (*U*^el^). Through interaction potentials for specific AAs with the NP, the overall interaction potential between the NP and the complete protein (*U*_p−NP_) is expressed in a pairwise additive manner:


[1] $$\begin{array}{c} U_{\text{p-NP}}=\sum_{i=1}^{N_{\text{AA}}}U_{i}\left(d_{i}\left(\theta ,\phi \right)\right)\\ =\sum_{i=1}^{N_{\text{AA}}}U_{i}^{\text{el}}\left(d_{i}\left(\theta ,\phi \right)\right)+\sum_{i=1}^{N_{\text{AA}}}U_{i}^{{\text{nb}}_{\text{s}}}\left(d_{i}\left(\theta ,\phi \right)\right)\\ +\sum_{i=1}^{N_{\text{AA}}}U_{i}^{{\text{vdW}}_{\text{l}}}\left(d_{i}\left(\theta ,\phi \right)\right). \end{array}$$

The potential *U*_p−NP_ depends on the distance *d_i_* between the centers of mass of the NP and each AA in the protein. This distance is determined by the protein’s orientation with respect to the NP’s surface, which is defined by two rotational angles (ϕ, θ) relative to the protein’s initial orientation. This initial orientation is set by performing a principle axis transformation such that the axis associated with the smallest moment of inertia is aligned to the *z* axis and the second smallest to the *y* axis, that is, the *z* axis is now typically associated with the greatest extent of the protein. Since this does not uniquely specify the orientation, further rotations of 180° are then applied if necessary such that the electric dipole moment is positive along these two axes. This produces a convenient reference state by which other orientations are defined. The specific orientation (ϕ, θ) is generated by applying a rotation of −ϕ around the *z* axis followed by a rotation of 180° − θ around the *y* axis. The short-range surface non-bonded potentials are extracted from AWT-MetaD simulations, which were described in the section “All-atoms short-range interaction modelling results”. The Hamaker technique is used to approximate the long-range term that results from the vdW forces working through the aqueous medium between the NP core and the *i*-th AA. The electrostatic interaction between the NP and AA is represented by the screened Coulomb potential. More comprehensive information about the theoretical aspects of the UA model can be found in our previous publications [2,25,28,39–40]. The output of the UA simulations contains a collection of rotational configurations and their corresponding *E*(θ_k_,ϕ_l_) values. By employing Boltzmann averaging and weighting factors based on the potential energy as a function of distance for each angle, we calculate the average adsorption energy of these configurations. Using this approach, we evaluate the adsorption energies of the entire proteins on aluminum surfaces. To predict the three-dimensional (3D) structures of proteins, we utilize the I-TASSER (Iterative Threading ASSEmbly Refinement) 5.1 software [41], which uses the protein’s AA sequences as an input.

For this study, we have chosen six representative cow milk proteins and lactose, which constitute most of the non-fat milk solids. Table 1 displays properties of the chosen compounds. It includes their UniProt IDs, molecular weights, charges, and the number of AAs in each protein. The charge data was determined through the PROPKA method [42–43] at a pH of 7.0. We model the lactose molecule as a pair of glucose beads; it does not possess a UniProt ID or a count of AA residues. We estimated the concentration of each protein and lactose based on their weight fraction in milk and considering the fact that cow milk has 30–39 g/L of protein and 45–55 g/L of lactose in total. The molar mass of each protein was taken from AlphaFold database [44]. Following this, all proteins underwent a 50 ns equilibration in water using NVT and NPT ensembles.

**Table 1:** Characteristics of the selected milk proteins and lactose.

Abbreviation	UniProt ID	Compound name	MW^a^, Da	Charge, *e*	Res^b^	*C*^c^ [10^−4^], mol/L	*R*_g_^d^ [Å]

AS1C	P02662	αs1-casein	24528.00	−8.5	214	4	20.05
AS2C	P02663	αs2-casein	26018.69	4.5	222	1	40.81
BC	P02666	β-casein	25107.33	−4.5	224	4	22.53
ALAC	P00711	α-lactalbumin	16246.61	−5	142	0.9	15.01
BLAC	P02754	β-lactoglobulin	19883.25	−6	178	2	15.50
BSA	P02769	bovine serum albumin	69293.41	−4.5	607	0.1	27.69
LAC	—	lactose	342.3	0	—	1300	4.28

^a^Molecular weight, ^b^Number of residues, ^c^Concentrations [mol/L] of the molecules in milk that were used in KMC calculations, ^d^Radius of gyration of the biomolecules in Ångstrom.

The UA computations were conducted using nine different Al NPs with varying radii, namely 2, 5, 10, 20, 30, 40, 50, 80, and 100 nm, to investigate the influence of size and curvature on the adsorption energies. The results and detailed information on the calculation can be found in Supporting Information File 1, Figure S2 and Figure S3, which illustrate the variations in adsorption energies as a function of NP size. Within the range of 2–20 nm the binding energies of ALAC, BLAC, BC, and BSA show an initial increase on all surfaces, followed by a stabilization at larger NP sizes. In contrast, AS1C and AS2C exhibit a continuous rise in binding energy across the entire size spectrum, ranging from −48.0*k*_B_*T* at 2 nm to −281.09*k*_B_*T* at 100 nm for AS1C and −15.26*k*_B_*T* at 2 nm to −275.60*k*_B_*T* at 100 nm for AS2C, with AS2C exhibiting the most dramatic changes in binding energy as a function of size. This strong size dependence in binding energy for AS2C can be attributed to its rod-like 3D structure and the rigidity assumption in our model. As the size of the NP increases, AS2C can make more extensive contact with the surface. This increased contact area leads to enhanced binding affinity, resulting in the observed stronger binding across the size range. This is not the case for other proteins on the list as they are more compact and, therefore, reach the maximum number of contacts at relatively small NP sizes. Regarding the binding affinity rankings, for the smallest NPs (2 nm), the order from weakest to strongest is observed as AS2C, BSA, ALAC, BLAC, AS1C, and BC on Al(100), with similar rankings observed on Al(110) and Al(111) surfaces. However, for the largest (flattest) NPs (100 nm), the binding affinity ranking changes to ALAC, BLAC, BSA, BC, AS2C, and AS1C on Al(100), BC, ALAC, BLAC, BSA, AS2C, and AS1C on Al(110), and BLAC, ALAC, BC, BSA, AS2C, and AS1C on Al(111) (see Supporting Information File 1, Figure S2). In reality, protein structures are not rigid, allowing them to adapt to the surfaces upon immobilisation. This can potentially affect their binding behavior. This can be especially significant for caseins, as they belong to the group of flexible milk proteins with no tertiary structure. Globular milk proteins (lactoglobulin and lactalbumin) are expected to be less prone to this shortcoming of the UA model.

**Figure 7:**
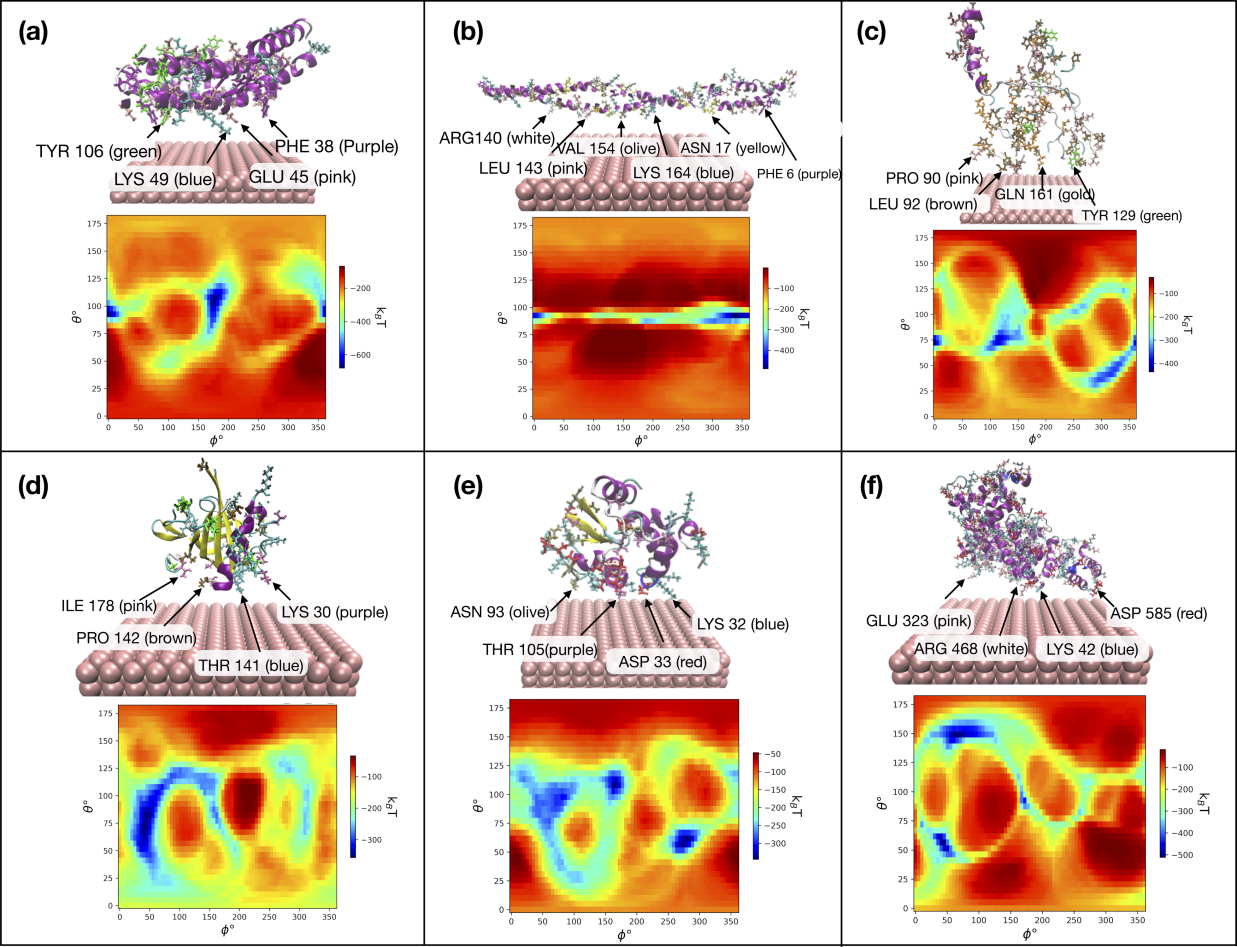
Adsorption energy heatmaps obtained from the UnitedAtom model and corresponding 3D representations of the interactions of (a) AS1C, (b) AS2C, (c) BC, (d) BLAC, (e) ALAC, and (f) BSA with Al(110) in the preferred orientations. The figure highlights the closest AAs to the surface of the material.

Figure 7 shows the output of the UA model for the selected milk proteins on aluminum NPs with a surface size of 80 nm with zeta potential −5 mV at pH 7.0. The heatmaps display the adsorption energies for all values of θ and ϕ. Blue areas with lower energies indicate more favorable orientations of the proteins. Each heatmap is accompanied by a 3D representation of the protein on the NP surface, with the AAs closest to the NP’s surface marked. The AAs that are most likely to make contact with the metal surfaces, according to analysis, are LYS, TYR, PHE, GLU, ARG, and ASP.

The rankings of protein adsorption on each aluminum surface are shown in Table 2, highlighting the variations in adsorption energies (*E*_ads_/*k*_B_*T*) and the particular protein–surface interactions (θ and ϕ in degrees). Moreover, the minimum distance (*r*_min_ in nm) indicates the closest approach of the protein to the aluminum surface during the adsorption process.

**Table 2:** Comparison of milk proteins’ binding affinities and orientations on Al(100), Al(110), and Al(111) with NP radius of 80 nm, derived from the UnitedAtom model and ordered by the binding strength on each surface.

Individual protein adsorption description on Al(100)				

Protein,	*E*_ads_/*k*_B_*T*	ϕ,°	θ,°	*r*_min_, nm

AS1C	−145.65	175	100	0.19
BC	−108.13	305	40	0.13
AS2C	−96.12	315	95	0.05
BSA	−91.11	45	60	0.11
BLAC	−67.35	65	90	0.19
ALAC	−49.12	125	35	0.20

Individual protein adsorption description on Al(110)				

Protein,	*E*_ads_/*k*_B_*T*	ϕ,°	θ,°	*r*_min_, nm

AS1C	−278.37	175	100	0.32
AS2C	−224.01	345	90	0.10
BSA	−173.77	40	60	0.23
BLAC	−157.70	50	95	0.28
ALAC	−155.17	70	90	0.29
BC	−132.52	0	70	0.20

Individual protein adsorption description on Al(111)				

Protein,	*E*_ads_/*k*_B_*T*	ϕ,°	θ,°	*r*_min_, nm

AS1C	−242.93	175	100	0.15
AS2C	−181.65	330	90	0.11
BSA	−137.46	45	60	0.13
BC	−131.93	140	110	0.15
ALAC	−125.76	75	90	0.17
BLAC	−113.39	45	75	0.20

The ranking of adsorption energies highlights the distinct adsorption behaviors of various proteins on different metal fcc surfaces. We can see that AS1C exhibits the highest adsorption energy on Al(100) and Al(111) surfaces, while on Al(110), AS1C, and AS2C show similar adsorption energies. In contrast, on metallic iron, AS1C consistently demonstrates the highest adsorption energy on Fe(100), Fe(110), and Fe(111) surfaces. This result reflects the size and shape of the AS1C protein, which allows it to make the largest number of contacts with the metal as compared to the other proteins. Regarding the most weakly bound proteins, on aluminum surfaces, ALAC consistently exhibits the lowest adsorption energy across all three surfaces, while BLAC shows slightly higher adsorption energies. In contrast, on iron surfaces, ALAC and BLAC demonstrate comparable adsorption energies, with ALAC exhibiting slightly lower energies on Fe(110) and Fe(111) surfaces [2]. We note that generally the binding of proteins to aluminum is weaker than to iron, which may be caused by the smaller lattice constant of fcc iron and higher density of surface atoms.

Supporting Information File 2, Table S2 reports the preferred orientations of all 820 milk proteins based on the lowest energy from the UnitedAtom output. In our investigation of these proteins, we focused on identifying the most strongly adsorbing proteins when exposed to Fe and Al. These proteins, including P19660, A6QP30, G3X745, F1MMI6, E1BBY7, A6QLY7, and Q9N2I2, demonstrated remarkable similarity in their binding behavior towards Fe(100) and Al(100) surfaces, E1BGJ4, A5D7M6, F1MMI6, A6QP30, G3X745, and F1N1C7 on Fe(110) and Al(110) surfaces, and F1MMI6 and E1B748 and A6QP30 on Fe(111) and Al(111) surfaces.

In the subsequent step, we predicted the composition of the milk protein layer at the aluminum surfaces. For this analysis, we consider the Al surface as a spherical NP with the protein layer uniformly adsorbed on its entire surface, forming the protein corona.

### Competitive adsorption and biomolecular corona

Kinetic Monte Carlo (KMC) simulations as implemented in the CoronaKMC tool [26] were employed to investigate competitive adsorption and to determine the composition of the protein corona. This method models adsorbates as hard spheres, which adsorb and desorb to the surface of the NPs, with different orientations of each protein treated as different potential adsorbates to allow for a more physically realistic model of corona formation for anisotropic proteins. In brief, a standard kinetic Monte Carlo routine is used to advance the simulation from one event, collision of an incoming adsorbate with the NP or desorption of an adsorbed species, to the next, with events occurring with a probability proportional to their rate. In the initial form of the model, adsorption is assumed to occur with unit probability if the incoming species does not overlap with any currently adsorbed species and fails to take place otherwise. We parameterize this model using adsorption and desorption rate constants extracted from UnitedAtom results as described previously [16,45]. In brief, each potential adsorbate (e.g., a small molecule or a particular orientation of a protein) is projected onto the surface of the NP and a convex hull procedure used to estimate the area of the NP occupied by that adsorbate, *A_i_*. The adsorbate is then assigned an effective radius *R_i_* such that a sphere projected onto the NP would produce the same radius [16]. The per-site adsorption rates are calculated using kinetic theory for the rate of collisions between two spheres in solution, normalized by the number of binding sites for that protein,


[2] $$k_{\text{a}}=\frac{A_{i}}{4\pi R_{\text{NP}}^{2}}\left[4\pi DN_{\text{A}}\left(R_{\text{NP}}+R_{i}\right)\right],$$

where *R*_NP_ is the radius of the NP, *N*_A_ is Avogadro’s number, *R*_A_ is the effective adsorbate radius, *D* is the pair diffusion coefficient given by


[3] $$D=\frac{k_{\text{B}}T}{6\eta }\left(R_{\text{NP}}^{-1}+R_{A}^{-1}\right),$$

taking the viscosity η = 8.9 × 10^−4^ Pa·s. We employ SI units in the above calculation, noting that *k*_a_ must then be multiplied by 1000 to convert from units m^3^·mol^−1^ to L·mol^−1^. Desorption rates are found by requiring that 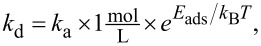 where *E*_ads_ is the value obtained for that orientation using UnitedAtom [45]. A concentration is then assigned to the adsorbate based on the bulk concentration of that adsorbate, weighted by the relative abundance of that orientation of the adsorbate if necessary. This means that for protein *i* with a bulk concentration of *C_i_* and a set of orientations θ*_k_*, an orientation θ*_j_* is assigned a concentration


[4] $$C_{i,j}=C_{i}\frac{\sin\theta_{j}}{\sum_{k}\sin\theta_{k}}$$

to ensure that orientations are correctly weighted and the total concentration summed over orientations is correctly reproduced. Scripts to automate this parameterization based on UA output and adsorbate structure files are available as part of the UnitedAtom repository [26].

We further analyze the results for adsorption of milk components obtained from KMC simulations, specifically focusing on the mean absolute and relative abundance of proteins (10^−3^ nm^2^) adsorbed on Al surfaces per unit area (nm^2^). Table 3 shows the abundances of proteins and lactose on Al surfaces.

**Table 3:** Mean amounts of proteins adsorbed on Al surfaces per unit area: number concentration (per nm^2^) and mass abundance obtained from KMC simulations with NPs of radius 80 nm. These calculations have been done using the KMC method with displacements.

	Al(100)	Al(100)	Al(110)	Al(110)	Al(111)	Al(111)

Protein	*N*_ads_ [10^−3^, nm^−2^]	*M*_ab_, %	*N*_ads_ [10^−3^, nm^−2^]	*M*_ab_, %	*N*_ads_ [10^−3^, nm^−2^]	*M*_ab_, %

AS1C	12.26	57.16	16.70	67.82	27.21	83.19
BC	4.45	21.24	3.38	14.07	1.91	5.84
BLAC	2.91	10.99	2.97	9.79	1.00	2.43
LAC	96.59	6.28	89.13	5.05	84.50	3.62
ALAC	1.14	3.51	1.13	3.05	1.84	3.60
AS2C	0.11	0.55	0.04	0.16	3.00	1.09
BSA	0.02	0.25	0.00	0.05	0.02	0.21

The simulations were performed using NPs with a radius of 80 nm, and the results are collected in Table 3. It presents the number concentration and mass abundance of proteins adsorbed on three different Al surfaces, namely Al(100), Al(110), and Al(111). Each protein’s adsorption behavior is quantified in terms of its number concentration (expressed in units of 10^−3^ nm^−2^) and mass abundance (represented as a percentage of the total adsorbed mass). These calculations were performed utilizing the most recent KMC method modifications, including an alternative mode in which the acceptance–rejection criteria for incoming adsorbates are altered to allow replacement of pre-existing adsorbates. We should note that Al(111) has the lowest energy of all three surfaces, according to the Materials Project data, so we expect the adsorption profile in real systems to be similar to that predicted for Al(111).

**Figure 8:**
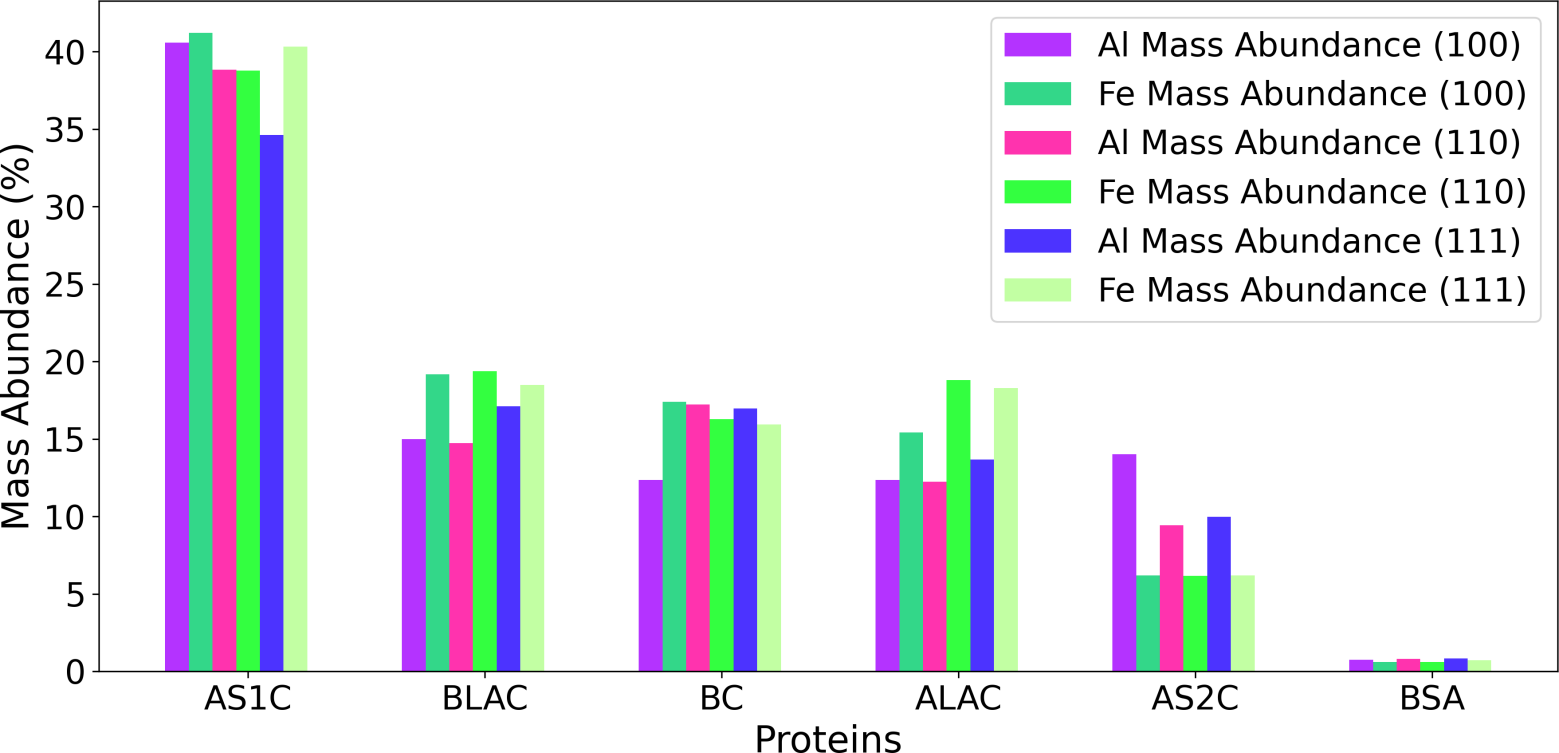
Mass abundance of proteins on Al and Fe surfaces (100, 110, and 111) using the original KMC approach without molecular displacements and a NP radius of 80 nm.

We also compared the protein composition in the corona on aluminum and iron [2], obtained in our previous work using the original KMC approach without molecular displacements. This comparison is shown in Figure 8. AS1C exhibited the highest abundance on both iron and aluminum among the studied proteins, indicating a strong affinity for both metals with both KMC methods as well as its high number concentration in solution. The following AS1C, BC, BLAC, and ALAC also showed fairly equal abundances on the surfaces of iron and aluminum. In contrast, BSA displayed the lowest abundance on both metals because of its larger size and the relatively low molar fraction in milk as compared with other proteins. Figure 8 shows the mass abundance of each protein on both aluminum (Al(100), Al(110), and Al(111)) and iron (Fe(100), Fe(110), and Fe(111)) surfaces. We can also observe that AS1C, BLAC, and ALAC display significantly enhanced presence on Fe surfaces in contrast to Al. Conversely, AS2C shows greater adsorption on Al surfaces as compared to Fe. Overall, we expect a somewhat different corona formed on these metallic surfaces.

Real-life organic media do not consist only of proteins, but they also include many other molecules, for example, sugars and other organic compounds that may bind to NPs along with proteins. It can reasonably be assumed that these molecules may alter both the kinetics and equilibrium state of the corona and, moreover, may play a role in biological outcomes. Thus, it is of interest to include these small molecules in the corona simulation to not only gain further insight into this particular case of aluminum in milk, but also to establish a methodology by which more general molecules can be included in these simulations. We choose lactose as a prototypical example of a small molecule capable of binding to NPs, since it is present at a high concentration in milk. We model the lactose molecule as a pair of glucose beads separated by a distance determined by the equilibrium structure of lactose. Although this is not completely rigorous, it demonstrates how the UnitedAtom software can be adapted to model larger molecules other than proteins using the same fragment-based approach. To avoid the need to run a time-consuming parameterization protocol based on metadynamics simulations, we produce PMFs for the glucose bead using a machine-learning technique (PMFPredictor) trained on previous metadynamics results [38]. For the lactose molecule, each constituent glucose bead is assigned a charge of 0, and the Hamaker term is neglected because of the small size of these beads. Following this parameterization, the coarse-grained lactose molecule is processed identically to proteins using the same automated pipeline, that is, UnitedAtom is run to produce a table of orientation-specific binding energies. These are mapped to rate constants for adsorption and desorption. We stress that this procedure is sufficiently generic that essentially arbitrary organic molecules can be included in the simulation by performing a fragment-based decomposition, generating PMFs via traditional or machine-learning approaches, and constructing a coarse-grained representation for input to UA. To simplify this procedure for more complex molecules, we have developed a Python script (MolToFragments.py) employing RDKit [46] to automate splitting larger molecules into suitable fragments and producing coarse-grained input files suitable for UnitedAtom and included it in this repository [26].

The addition of lactose (or other small molecules) to the corona simulation poses a challenge for the form of the CoronaKMC algorithm previously employed because of the high concentration and very small binding area of this small molecule relative to proteins [16,45]. As a consequence of these factors, the original form of the algorithm results in rapid coverage of the NPs with a very large quantity of lactose. This greatly increases the required computational time, which scales as 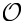
(*N*^2^) for *N* adsorbed particles. Moreover, in this original form of the model, a single adsorbed lactose molecule inhibits the adsorption of a large protein, no matter how strongly the protein may adsorb. To counteract these issues, the following features were added to the new version of the CoronaKMC software. First, we implemented a method to accelerate the simulation by adjusting rate constants for quasi-equilibriated processes (e.g., the adsorption of lactose) according to the methodology of Dybeck and co-workers [47]. Second, we added an optional mode in which the acceptance–rejection criteria for an incoming adsorbate are modified such that an incoming adsorbate is no longer immediately rejected if it overlaps with a pre-existing adsorbate. Instead, the incoming adsorbate is accepted with a probability *p* given by,


[5] $$p\left(\Delta E\right)=\frac{\exp\left[-\Delta E/k_{\text{B}}T\right]}{1+\exp\left[-\Delta E/k_{\text{B}}T\right]},$$

where Δ*E* is the difference in energy between the two states,


[6] $$\Delta E=E_{\text{ads}}-\sum_{j}E_{j},$$

where *j* is the set of all adsorbed particles that would overlap with this particle, taking Δ*E* = *E*_ads_ if no overlaps are found. If the adsorbate is accepted, then all the overlapping particles are removed from the NP. We note that this breaks the principle of detailed balance in that it allows for the replacement of a set of adsorbates by a single molecule, but does not allow for the converse in which a set of incoming molecules can displace an adsorbate. We justify this neglect on the basis that the required event of multiple simultaneous collisions on a single target would occur so rarely that it would essentially not be sampled in the course of a simulation. The probabilistic acceptance to regions of the NP without explicit adsorbates present effectively multiplies the adsorption rate by a factor of *p*(*E*_ads_). Thus, to maintain the same equilibrium constant, we must multiply the desorption rate by this same factor, noting that this correction is only significant for very weakly adsorbing particles with *E*_ads_ 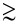 −3*k*_B_*T*. This methodology does not treat adsorption of water to the NP explicitly. Instead, it is assumed that all binding energies are defined relative to the adsorption of water, which is assigned an affinity *E*_ads_ = 0*k*_B_*T*, and that the concentration of water is sufficiently high such that any region of the NP without an explicit adsorbate can be assumed to be covered in water.

The results of simulations obtained with the updated CoronaKMC (i.e., including the molecule displacement) are shown in Table 3, and they suggest a notable variation in the abundances of proteins and lactose among different Al crystallographic orientations. Notably, on all surfaces studied, AS1C and BC consistently exhibited the highest protein abundances, while BLAC, LAC, and ALAC demonstrated moderate adsorption levels. In contrast, AS2C and BSA consistently displayed the lowest adsorption among the proteins considered in our simulations. Furthermore, when considering different Al facets, it is evident that the (110) surface consistently exhibited the weakest average adsorption across all proteins. When the displacement is allowed, AS1C gains much more space in the corona by replacing other proteins, mostly BLAC, ALAC, and AS2C.

**Figure 9:**
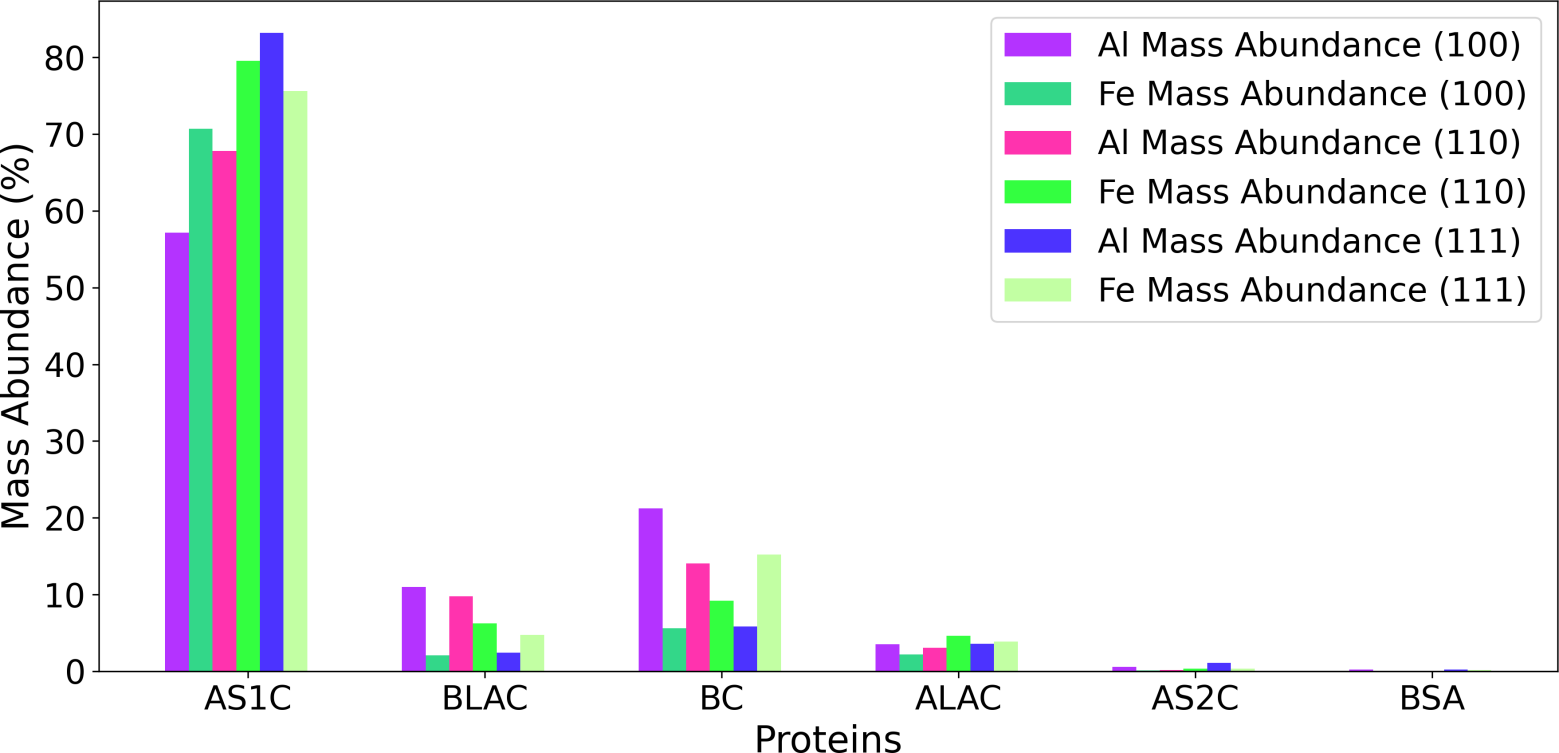
Mass abundance of proteins on Al and Fe surfaces (100, 110, and 111) using the KMC model with molecule displacement and a NP radius of 80 nm.

Figure 9 presents a comparison between the protein abundances in the corona on Al and Fe obtained using the enhanced version of the KMC algorithm with molecular displacements. As discussed earlier, this improved algorithm addresses computational efficiency concerns and more accurately represents long-term scenarios during protein corona formation. As shown in the Figure, these algorithmic improvements have a profound impact on the mass concentration of milk proteins on metallic surfaces, particularly on iron. In the original algorithm (Figure 8), proteins showed comparable mass abundances on both metals. However, the enhanced algorithm reveals a distinct change in the adsorption behavior of the AS1C protein on Fe and Al surfaces, characterized by a substantial increase in mass concentration compared to other proteins. The data in Table 3 show that in terms of mass abundance lactose ranks fourth among the corona components (see Supporting Information File 1, Figure S3). As compared to the algorithm without displacement [2], the protein abundance ranking on iron (NP radius 80 nm) surfaces changes to AS1C ≫ BC ≥ BLAC ≥ ALAC *>* AS2C ≈ BSA. A comparable affinity ranking is also now observed for aluminum surfaces (80 nm) studied in current work: AS1C ≫ BC ≥ BLAC ≥ ALAC *>* AS2C ≈ BSA.

## Conclusion

In this work, we applied a multiscale computational model to study the adsorption of milk solids on the metallic surfaces of aluminum, widely used in food processing/packaging. The milk model contained the six most common milk proteins and lactose. To account for the size differences of selected milk constituents, we used an improved competitive adsorption algorithm that can potentially achieve a realistic description of biocorona formation processes with diverse adsorbates (e.g., for predicting an eco-corona).

Our computational model predicts strong binding of milk proteins to pure aluminum surfaces, which is in agreement with our previous observations for metallic iron surfaces [2]. For aluminum, we also found that AS1C and AS2C exhibited the strongest binding to the metal, followed by BSA, BC, BLAC, and ALAC, which displayed weaker adsorption. We also found similar protein abundances in the corona for the two metals demonstrated by KMC simulation results. AS1C dominates the adsorption as the most abundant protein on aluminum surfaces, with BSA being the least abundant. We found a small difference in the predicted corona content between the two metals: BC and BLAC prefer Al(100) and Al(110) to iron, while AS1C prefers Fe(100) and Fe(110) over aluminum.

Although the adsorption energy regulates the interaction strength between proteins and surfaces, the mass concentration of proteins in the solution has a major effect on the amount of protein adsorbed onto the surface. Expanding the milk model by adding lactose into the mix did not alter the ranking of protein abundance in the corona. Despite the high concentration in the milk, lactose does not exceed the mass abundance of specific proteins such as AS1C due to its small size. In our model, it essentially forms a thin monolayer on the surface.

Overall, our freely accessible multiscale computational model [26] allows us to make predictions of the binding strength, preferred orientations, and relative abundance of the specified molecules on the specified material surfaces or NPs and, thus, gives an insight into the mechanisms of bionano interaction. We can compare different materials in terms of the protein binding affinity and corona content and optimize the processes in food and chemical industry. The presented methodology can be easily extended to other molecules, materials, and contexts involving the bionano interface such as environmental safety, health, medical devices, or toxicology.

## Supporting Information

Table S1: Adsorption free energies for each SCA on Al surfaces; Figure S1: Water density profiles for aluminum slabs: (a) Al(100), (b) Al(110), (c) Al(111), Figure S2: Influence of the NP size on the adsorption energies; Figure S3: Milk molecules ranking based on mass abundance in the corona, Figure S4: Example of AlNP size-dependent interaction of ALAC: (a) 2 nm, (b) 5 nm, (c) 10 nm, (d) 20 nm, (e) 40 nm, (f) 50 nm, (g) 80 nm, (d) 100 nm; Figures S5–S10: Comparison of interaction of AS1C, AS2C,β-casein, ALAC, BLAC, BSA, with different Al fcc surfaces: (a) Al(100), (b) Al(110), (c) Al(111); Table S2: Description of 820 milk proteins interaction with Al (100, 110, 111) based on the lowest energy values of the adsorption heatmaps.

File 1 Supporting material.

File 2 820-Milk-protein-table.
