# Supplementary material for: Multiscale modelling of biomolecular corona formation on metallic surfaces
Source: Beilstein J Nanotechnol. 2024 Feb 13;15:215–29. doi: 10.3762/bjnano.15.21 (PMC10877083; doi:10.3762/bjnano.15.21)
Supplement: File 1 — Supporting material. [file Beilstein_J_Nanotechnol-15-215-s001.pdf]

BEILSTEIN JOURNAL OF NANOTECHNOLOGY

Supporting Information

for

Multiscale modelling of biomolecular corona formation on metallic surfaces

Parinaz Mosaddeghi Amini, Ian Rouse, Julia Subbotina and Vladimir Lobaskin

Beilstein J. Nanotechnol. **2024,** *15,* 215–229. doi:10.3762/bjnano.15.21

Supporting material

License and Terms: This is a supporting information file under the terms of the Creative Commons Attribution License (https://creativecommons.org/licenses/by/4.0). Please note that the reuse, redistribution and reproduction in particular requires that the author(s) and source are credited and that individual graphics may be subject to special legal provisions.
The license is subject to the *Beilstein Journal of Nanotechnology* terms and conditions: (https://www.beilstein-journals.org/bjnano/terms)

Contents

1 Adsorption Free Energies for each SCAs on Al surfaces S3

2 Water density profiles for Aluminium slabs S4

3 Influence of the NP size on the protein adsorption energies S5

4 Milk molecules ranking based on mass abundance in the corona S6

5 Example of Al NP size-dependent interaction of α-lactalbumin S7

6 Comparison of interaction of proteins with different Al fcc surfaces S8

6.1 αs1-casein S8
6.2 αs2-casein S9
6.3 β- casein S10
6.4 α-lactalbumin S11
6.5 β-lactoglobulin S12
6.6 Bovine Serum Albumin S13

1 Adsorption Free Energies for each SCAs on Al surfaces

Table S 1. Adsorption Free Energies (in k_BT) for Side Chain Analogues (SCA) on the Al(100-110-111) Surfaces from Adaptive Well-Tempered Metadynamics (AWT-MetaD) simulations

SCA	Class^a	%^{b}	T-group^c	Charge	E_{min}Al-100	E_{min}Al-110	E_{min}Al-111
ALA	H	7.5	CH_3	0	−2.30	−1.76	−3.33
ARG	C^+	5.2	NH_2	+1	−41.46	−56.26	−55.76
ASP	C^-	5.2	O	−1	−9.34	−20.90	−25.57
ASN	P	4.6	NH_2	0	−19.06	−29.89	−31.52
CYS	P	1.8	CH	0	−5.44	−15.24	−12.77
GAN	P	6.3	COOH	0	−30.58	−30.95	−38.62
GLN	P	4.1	NH_2	0	−25.01	−30.31	−33.93
GLU	C^-	6.3	O	−1	−12.44	−27.08	−23.59
GLY	P	7.1	CH_2	0	−25.91	−37.84	−37.88
HIS	A	2.2	NH	0	−19.88	−37.26	−45.64
HIE	A	2.2	NH	0	−26.15	−32.76	−43.86
ILE	H	5.5	CH_3	0	−2.52	−32.84	−22.78
LEU	H	9.1	CH_3	0	−16.79	−22.59	−5.21
LYS	C^+	5.8	NH_2	+1	−17.64	−30.60	−28.54
MET	H	2.8	NH_2	0	−13.38	−28.72	−24.81
PHE	A	3.9	CH	0	−20.58	−26.02	−42.45
PRO	P	5.1	CH_2	0	−51.68	−50.13	−55.38
SER	P	7.4	OH	0	−6.93	−19.52	−12.65
THR	P	6.0	OH	0	−5.21	−19.51	−12.65
TRP	A	1.3	NH	0	−47.44	−45.23	−63.32
TYR	A	3.3	OH	0	−22.43	−34.29	−56.94
VAL	H	6.5	CH_3	0	−2.83	−17.50	−13.41

^{a} H = hydrophobic, P = polar, A = aromatic, and C = charged.,^{b}the percentage of AAs composition in proteins ([1]), ^{c} Terminating group in SCA.

2 Water density profiles for Aluminium slabs

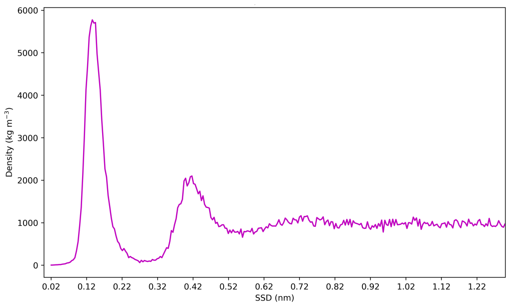

(a) Al-100

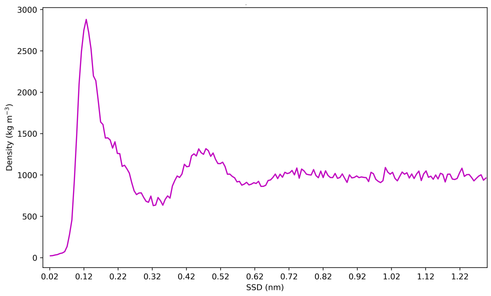

(b) Al-110

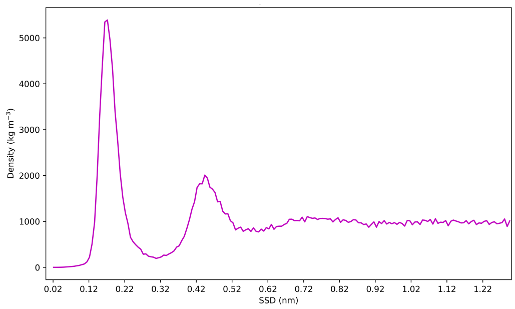

(c) Al-111

Figure S 1. Water density profile obtained from MD simulation for (a) Al-100, (b) Al-110 and (c) Al-111. The profiles exhibit distinct peaks indicating the presence of water layers near the surface. The first peak, observed at approximately 0.15-0.18 nm, corresponds to the formation of the first water layer adjacent to the aluminum surface. The second peak, occurring around 0.42-0.48 nm, represents the presence of a second water layer.

3 Influence of the NP size on the protein adsorption energies

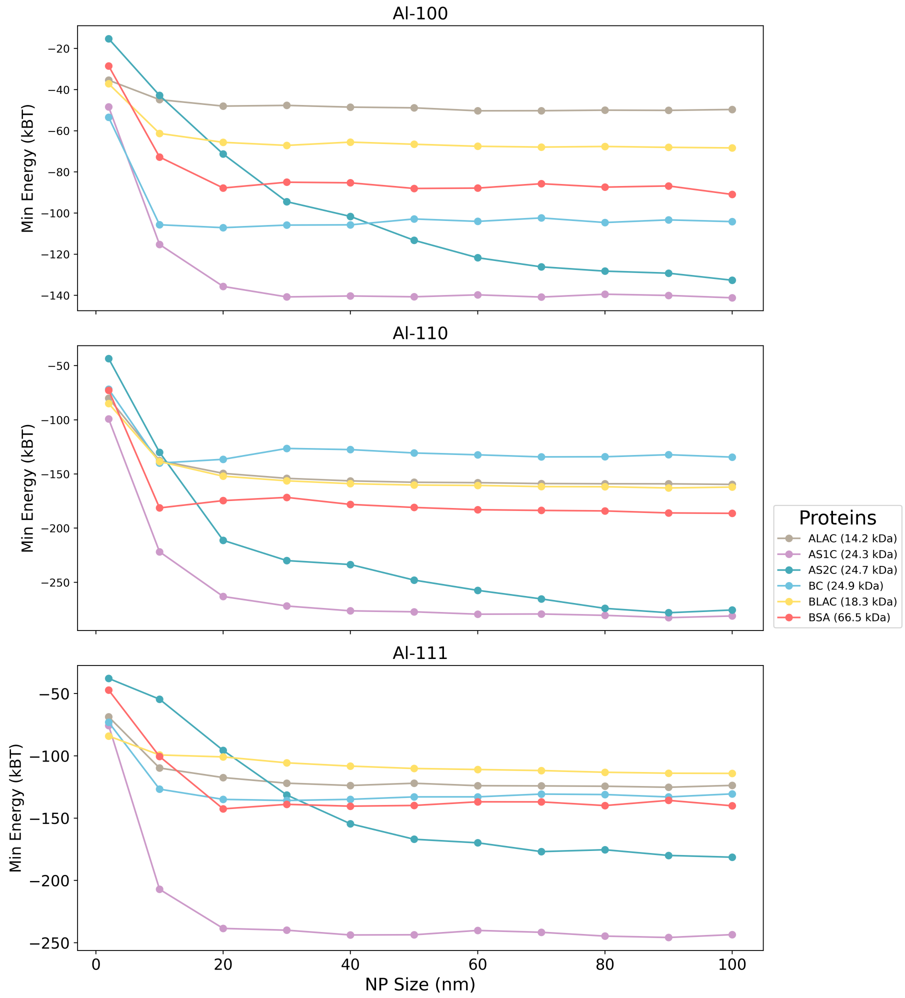

Figure S 2. Energy of adsorption (k_BT) as a function of NP size (R, nm) for six selected milk proteins on Al-100, Al-110, and Al-111 obtained from United Atom model.

4 Milk molecules ranking based on mass abundance in the corona

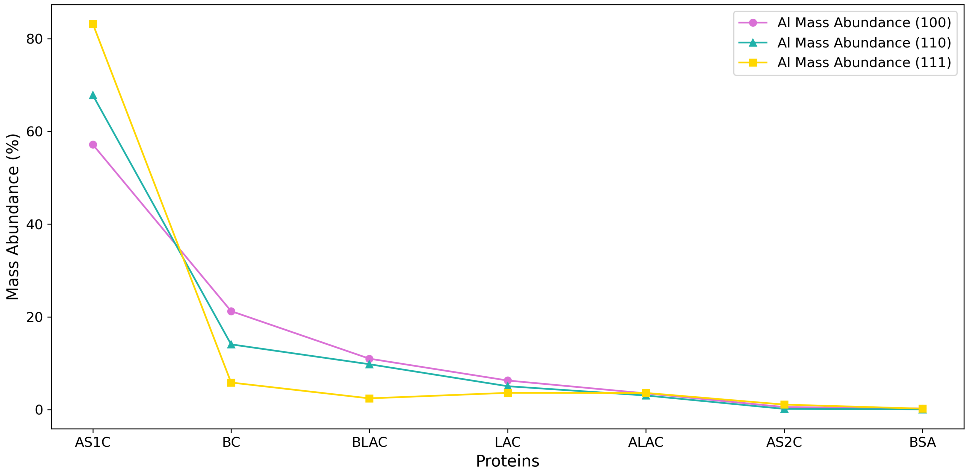

Figure S 3. Protein and lactose mass abundance in the corona on Al surfaces as obtained using the KMC model with molecular displacements, with lactose emerging as the fourth most prevalent compound. The lines are guides to the eye.

5 Example of Al NP size-dependent interaction of α-lactalbumin

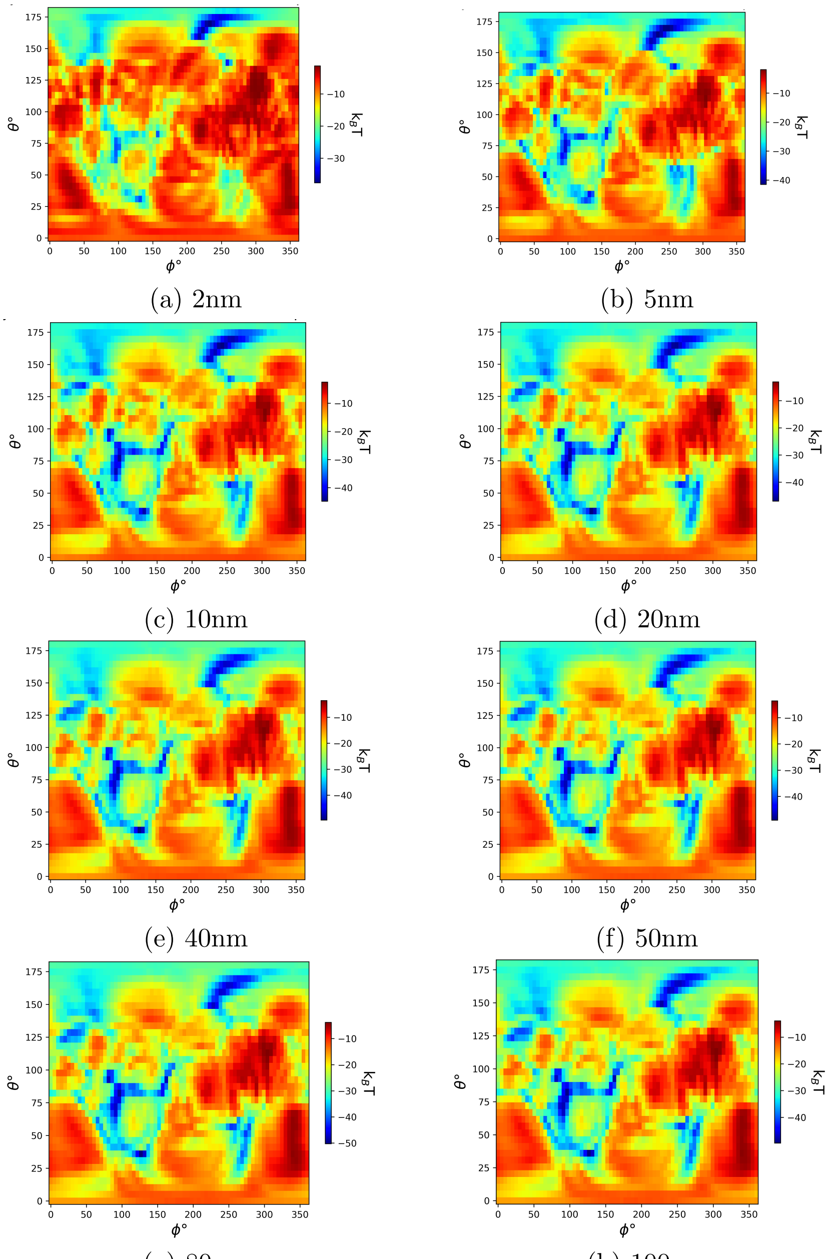

(a) 2nm
(b) 5nm
(c) 10nm
(d) 20nm
(e) 40nm
(f) 50nm
(g) 80nm
(h) 100nm

Figure S 4. The heatmap evolution based on Al NP of radius 100 nm for α-lactalbumin obtained from United Atom model.

6 Comparison of interaction of proteins with different Al fcc surfaces

6.1 αs1-casein

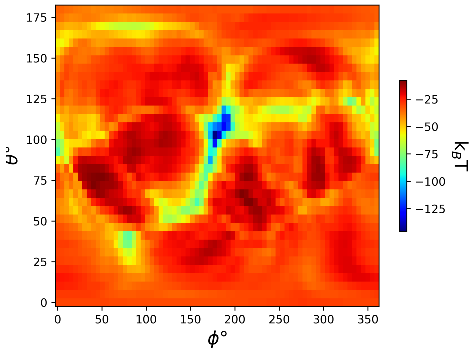

(a) Al-100

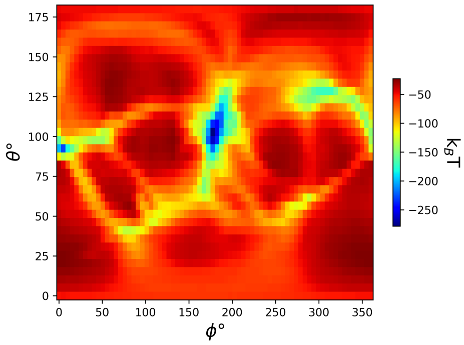

(b) Al-110

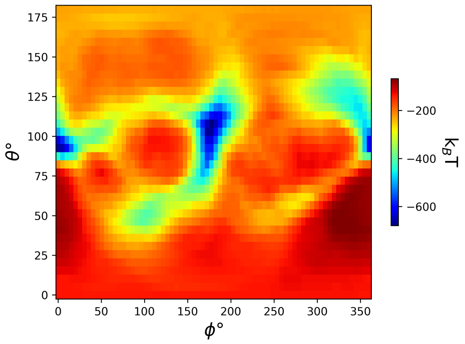

(c) Al-111

Figure S 5. Adsorption energy heatmaps for αs1-casein on (a) Al-100, (b) Al-110, (c) Al-111 with NP of radius 30 nm obtained from United Atom model

6.2 αs2-casein

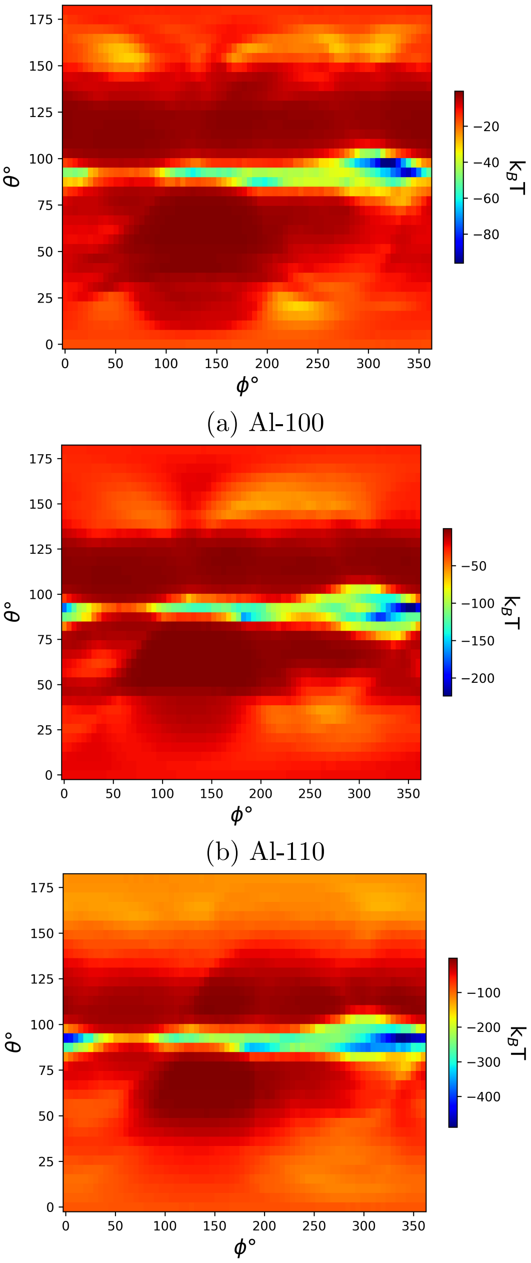

(a) Al-100

(b) Al-110

(c) Al-111

Figure S 6. Adsorption energy heatmaps for αs2-casein on (a) Al-100, (b) Al-110, (c) Al-111 with NP of radius 30 nm obtained from United Atom model

6.3 β- casein

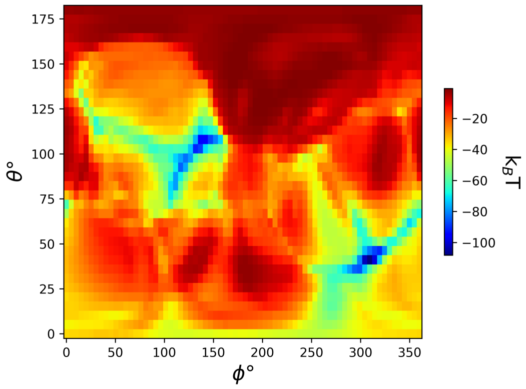

(a) Al-100

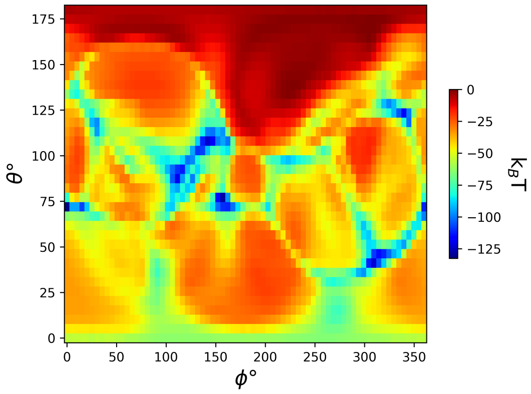

(b) Al-110

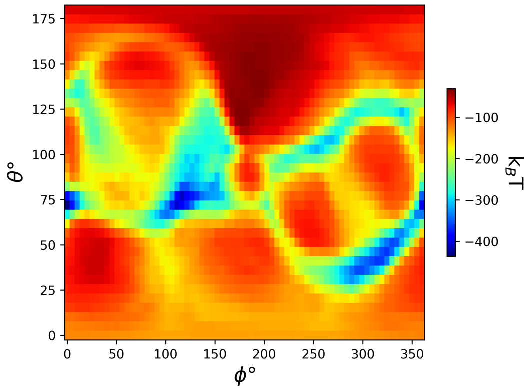

(c) Al-111

Figure S 7. Adsorption energy heatmaps for β-casein on (a) Al-100, (b) Al-110, (c) Al-111 with NP of radius 30 nm obtained from United Atom model

6.4 α-lactalbumin

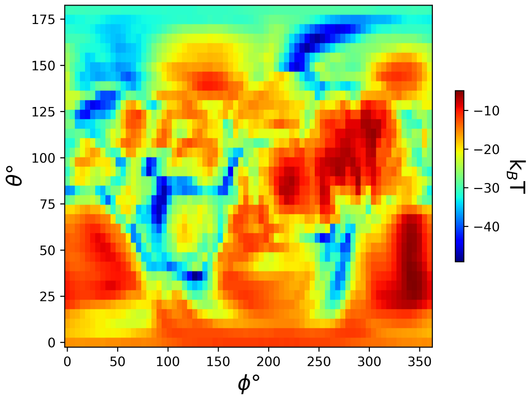

(a) Al-100

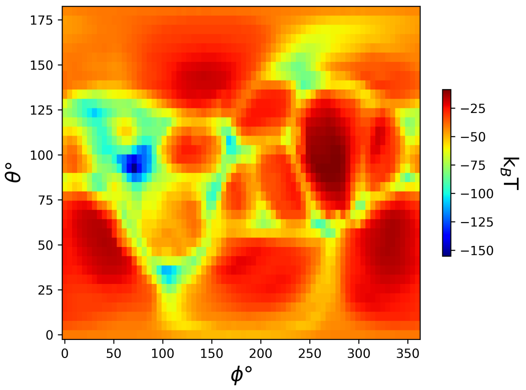

(b) Al-110

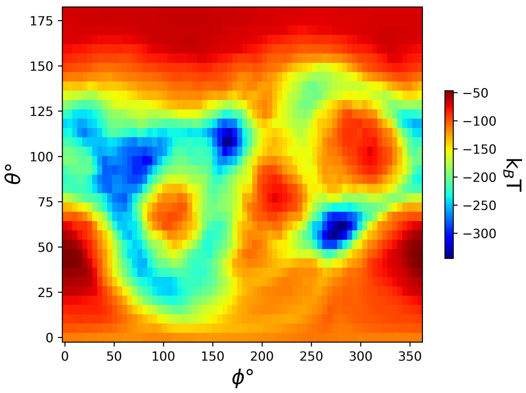

(c) Al-111

Figure S 8. Adsorption energy heatmaps for α-lactalbumin on (a) Al-100, (b) Al-110, (c) Al-111 with NP of radius 30 nm obtained from United Atom model

6.5 β-lactoglobulin

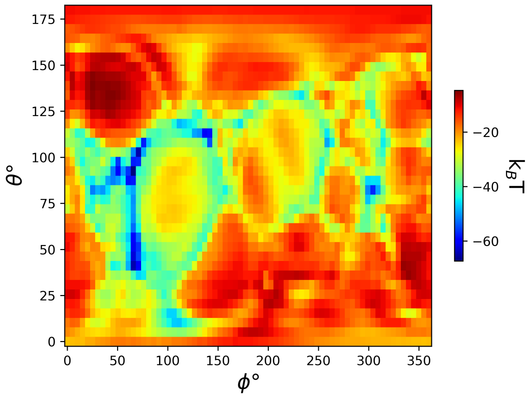

(a) Al-100

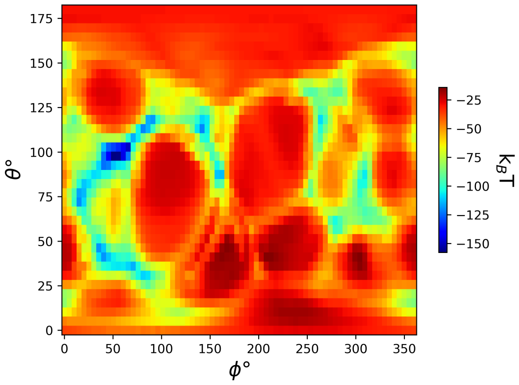

(b) Al-110

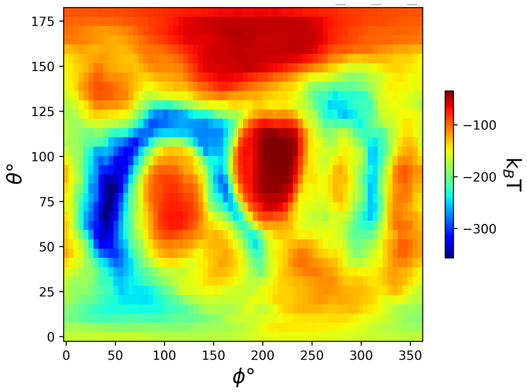

(c) Al-111

Figure S 9. Adsorption energy heatmaps for β-lactoglobulin on (a) Al-100, (b) Al-110, (c) Al-111 with NP of radius 30 nm obtained from United Atom model

6.6 Bovine Serum Albumin

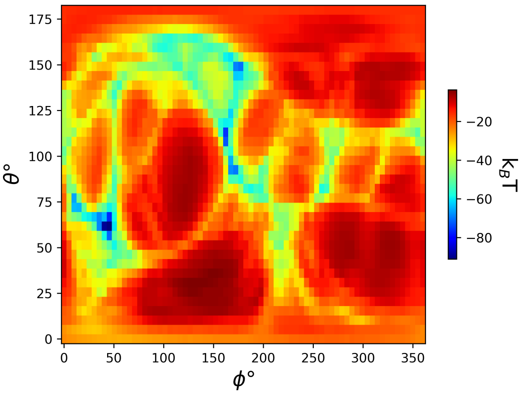

(a) Al-100

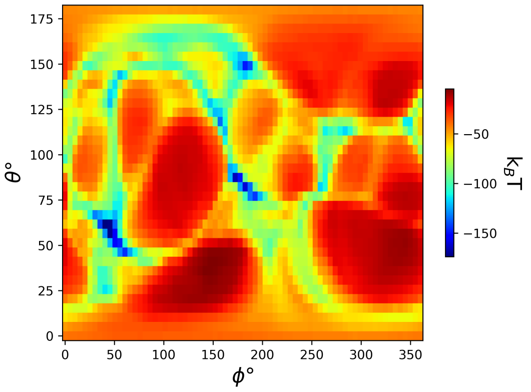

(b) Al-110

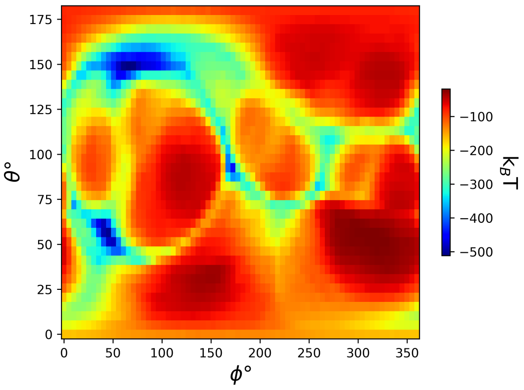

(c) Al-111

Figure S 10. Adsorption energy heatmaps for Bovine Serum Albumin on (a) Al-100, (b) Al-110, (c) Al-111 with NP of radius 30 nm obtained from United Atom model

References

[1] Georges Trinquier and Yves-Henri Sanejouand. “Which effective property of amino acids is best preserved by the genetic code?” In: *Protein engineering* 11.3 (1998), pp. 153–169.
