# Supplementary material for: Multiscale modelling of biomolecular corona formation on metallic surfaces
Source: Beilstein J Nanotechnol. 2024 Feb 13;15:215–29. doi: 10.3762/bjnano.15.21 (PMC10877083; doi:10.3762/bjnano.15.21)
Supplement: File 2 — 820-Milk-protein-table. [file Beilstein_J_Nanotechnol-15-215-s002.pdf]

BEILSTEIN JOURNAL OF NANOTECHNOLOGY

Supporting Information

for

Multiscale modelling of biomolecular corona formation on metallic surfaces

Parinaz Mosaddeghi Amini, Ian Rouse, Julia Subbotina and Vladimir Lobaskin

Beilstein J. Nanotechnol. **2024,** *15,* 215–229. doi:10.3762/bjnano.15.21

820-Milk-protein-table

License and Terms: This is a supporting information file under the terms of the Creative Commons Attribution License (https://creativecommons.org/licenses/by/4.0). Please note that the reuse, redistribution and reproduction in particular requires that the author(s) and source are credited and that individual graphics may be subject to special legal provisions.
The license is subject to the *Beilstein Journal of Nanotechnology* terms and conditions: (https://www.beilstein-journals.org/bjnano/terms)

Table S2: Description of 820 milk proteins interaction with Al (100, 110, 111) based on the lowest energy values of the adsorption heatmaps.

	Al-100				Al-110				Al-111			
**Milk Protein ID**	**phi**	**theta**	**min-energy (KT)**	**min-dist (nm)**	**phi**	**theta**	**min-energy (KT)**	**min-dist (nm)**	**phi**	**theta**	**min-energy (KT)**	**min-dist (nm)**
**Q148H8**	35	125	-89.89	0.12	215	145	-173.05	0.24	0	110	-192.54	0.08
**Q9XSG3**	85	65	-88.68	0.10	270	45	-249.33	0.26	260	150	-208.62	0.05
**E1BEV7**	215	115	-129.33	0.14	65	60	-259.86	0.20	35	125	-158.29	0.16
**F2Z4F5**	255	55	-115.52	0.12	255	55	-235.16	0.22	40	85	-202.56	0.16
**P33097**	160	135	-102.95	0.18	155	135	-227.71	0.28	295	170	-153.25	0.09
**A7YWG4**	120	65	-90.80	0.17	120	65	-168.36	0.27	155	65	-166.96	0.07
**F1MGS9**	265	130	-95.98	0.02	265	135	-200.10	0.11	210	95	-186.36	0.15
**E1B991**	355	145	-116.24	0.03	355	145	-339.78	0.06	345	40	-182.70	0.11
**Q2HJ81**	210	90	-102.30	0.19	210	95	-198.42	0.35	325	100	-129.17	0.19
**A3KN30**	350	65	-108.65	0.15	345	35	-212.13	0.21	20	65	-151.41	0.04
**A1A4K3**	185	40	-85.07	0.19	130	140	-211.19	0.15	220	95	-145.38	0.17
**P00735**	270	55	-130.95	0.12	275	55	-226.39	0.15	270	55	-186.16	0.11
**P31098**	205	90	-73.06	0.17	345	60	-180.24	0.23	205	50	-120.86	0.16
**O62830**	215	100	-86.58	0.18	250	45	-183.90	0.08	185	45	-155.49	0.16
**E1B898**	15	60	-105.08	0.10	25	75	-260.09	0.12	65	60	-183.40	0.11
**A6QPZ4**	110	125	-88.21	0.17	35	120	-175.14	0.25	275	45	-159.12	0.09
**Q3ZBZ8**	40	105	-136.90	0.13	40	85	-214.45	0.33	40	120	-139.22	0.11
**G3X7D2**	105	85	-109.01	0.20	155	70	-246.83	0.22	270	135	-147.40	0.05
**Q58DL9**	300	170	-113.26	0.05	295	160	-215.18	0.04	115	60	-140.07	0.17
**G3N0V2**	195	135	-103.69	0.05	205	145	-286.06	0.09	355	145	-209.49	0.04
**Q0VCN9**	0	115	-105.69	0.21	0	115	-227.73	0.30	255	55	-184.69	0.09
**Q9MZ06**	160	100	-86.78	0.21	285	75	-173.85	0.23	155	135	-165.45	0.16
**F1MC11**	195	55	-95.84	0.17	0	100	-219.92	0.09	20	55	-151.81	0.03
**E1BJL8**	35	85	-101.65	0.19	215	75	-210.31	0.27	145	170	-109.33	0.17
**Q09TE3**	140	40	-112.33	0.19	145	105	-246.16	0.14	340	55	-162.46	0.16
**E1B8A0**	5	60	-106.72	0.20	345	145	-206.72	0.21	245	65	-94.33	0.09
**E1BEC6**	75	145	-63.53	0.01	215	45	-159.61	0.11	170	115	-220.79	0.12
**P23805**	65	55	-95.16	0.19	140	105	-172.02	0.12	255	45	-163.54	0.15
**Q2KJJ8**	340	130	-123.56	0.19	280	135	-227.18	0.22	65	55	-154.68	0.16
**E1BI82**	355	135	-131.77	0.08	75	110	-220.88	0.25	200	125	-128.62	0.12
**P62992**	60	165	-131.94	0.19	125	165	-222.43	0.12	80	70	-158.90	0.17
**F1MME1**	250	75	-120.30	0.11	250	75	-219.14	0.24	70	55	-148.76	0.13
**A4FV69**	160	75	-82.76	0.20	215	90	-186.39	0.27	20	60	-190.74	0.16
**G3N156**	80	135	-101.15	0.14	75	145	-218.29	0.27	300	120	-178.92	0.11
**F1MSB7**	175	95	-91.83	0.13	175	95	-211.71	0.21	305	15	-128.81	0.10
**P28800**	80	75	-111.18	0.19	60	105	-210.05	0.20	280	120	-114.29	0.05
**Q29451**	170	115	-130.01	0.14	175	110	-265.73	0.21	285	115	-145.00	0.02
**Q0P5J6**	125	125	-65.14	0.15	195	125	-168.13	0.14	55	100	-139.94	0.16
**P28801**	295	110	-76.35	0.21	65	55	-185.92	0.32	115	165	-221.82	0.08
**Q2LGB2**	335	45	-93.08	0.20	55	50	-183.27	0.28	0	135	-198.92	0.13
**A6QNS6**	245	65	-57.44	0.08	245	70	-119.85	0.05	280	130	-166.56	0.14
**E1BPC9**	285	115	-97.75	0.02	285	115	-187.91	0.11	260	105	-132.56	0.14

**Q2HJD1**	275	45	-91.60	0.22	55	105	-175.75	0.26	220	45	-115.49	0.10
**F1MCX1**	115	165	-123.51	0.09	120	160	-241.99	0.09	320	130	-140.35	0.15
**O18836**	250	75	-84.62	0.08	250	80	-152.02	0.14	75	140	-173.29	0.06
**Q17QC7**	280	120	-76.95	0.00	340	90	-134.48	0.07	175	95	-155.23	0.09
**F1MUZ8**	70	145	-84.16	0.20	195	160	-190.63	0.25	250	75	-199.58	0.10
**Q58CS8**	20	60	-121.04	0.18	20	55	-216.78	0.25	60	165	-181.46	0.15
**F1N1I6**	300	120	-128.18	0.14	255	55	-223.32	0.21	145	45	-189.16	0.14
**F1N789**	20	55	-129.15	0.02	15	55	-239.64	0.08	60	100	-163.22	0.16
**P00743**	345	60	-105.17	0.15	195	80	-193.23	0.13	195	55	-168.39	0.14
**A5PKI3**	180	170	-81.34	0.23	105	45	-143.53	0.22	330	130	-145.13	0.16
**F1MSZ6**	200	60	-102.61	0.20	240	95	-199.79	0.25	255	45	-136.62	0.17
**E1BBX5**	95	65	-120.69	0.17	75	75	-247.09	0.22	175	60	-164.77	0.11
**E1BQ21**	185	75	-92.63	0.21	300	120	-174.47	0.27	305	145	-119.49	0.17
**B0JYP8**	100	90	-92.47	0.22	110	95	-173.78	0.28	130	140	-153.91	0.17
**Q9MYM4**	245	55	-110.43	0.12	240	75	-212.91	0.23	0	150	-157.65	0.11
**P50448**	0	45	-85.67	0.07	255	145	-157.21	0.22	80	135	-148.28	0.12
**Q2KJ63**	30	135	-132.22	0.08	30	130	-254.60	0.13	120	20	-138.32	0.18
**Q0IIG7**	335	75	-90.15	0.21	70	10	-179.47	0.19	20	20	-173.05	0.10
**E1B725**	335	110	-116.60	0.16	275	145	-209.84	0.24	290	85	-174.18	0.11
**Q95M12**	325	30	-135.52	0.12	335	25	-215.80	0.26	195	15	-197.84	0.14
**F1MNV5**	350	70	-95.06	0.13	325	90	-156.97	0.28	250	110	-160.57	0.11
**A6QR11**	0	0	-0.00	2.00	0	0	-0.00	2.00	50	120	-158.20	0.08
**P28783**	350	100	-85.82	0.17	60	105	-191.93	0.20	195	100	-152.24	0.08
**Q03763**	185	125	-47.98	0.00	190	130	-136.22	0.05	350	70	-143.51	0.10
**Q3ZBH5**	110	75	-92.78	0.20	340	20	-166.79	0.26	0	0	-0.00	2.00
**Q5E9Z9**	50	95	-51.66	0.21	110	70	-121.25	0.04	110	75	-98.84	0.01
**A5D960**	5	150	-98.07	0.15	330	115	-194.49	0.24	5	20	-118.29	0.17
**G5E513**	260	65	-83.98	0.15	260	65	-197.22	0.21	180	130	-91.99	0.13
**E1BF48**	130	140	-107.93	0.19	175	90	-177.13	0.30	50	110	-145.19	0.04
**Q3T169**	105	130	-100.10	0.18	165	55	-178.47	0.24	335	75	-138.72	0.17
**P62935**	345	35	-82.44	0.22	80	135	-160.08	0.31	30	130	-188.52	0.03
**F1MUB9**	255	45	-86.93	0.19	255	50	-207.36	0.27	330	30	-194.63	0.14
**F1MBV6**	265	100	-91.62	0.03	30	130	-195.01	0.04	160	130	-175.87	0.16
**Q29S21**	195	100	-89.68	0.09	200	135	-193.79	0.01	200	60	-146.14	0.16
**F1MF70**	195	15	-115.47	0.18	200	15	-219.26	0.18	80	75	-194.28	0.13
**E1B8B5**	250	110	-108.42	0.13	75	130	-187.78	0.24	110	95	-160.31	0.10
**F1ML49**	290	85	-110.80	0.12	240	45	-211.08	0.08	15	50	-105.80	0.14
**G3N2B8**	115	20	-82.27	0.21	130	35	-183.03	0.28	240	75	-170.20	0.12
**Q76LV1**	10	25	-100.42	0.03	15	25	-200.87	0.09	295	120	-144.20	0.16
**G3X7N4**	165	85	-106.01	0.16	160	85	-183.98	0.33	15	75	-136.54	0.17
**F1MY79**	30	55	-83.33	0.08	200	145	-211.76	0.18	290	90	-153.16	0.19
**A5D984**	40	25	-107.05	0.21	35	35	-206.17	0.17	330	120	-131.47	0.08
**F1MS40**	75	115	-94.19	0.12	70	110	-201.88	0.17	190	130	-120.81	0.06
**Q28133**	105	60	-99.80	0.20	110	65	-168.45	0.30	130	155	-159.27	0.01
**Q2KIW9**	125	105	-73.11	0.20	120	110	-156.75	0.25	255	90	-141.54	0.02
**F1MGK8**	180	105	-88.64	0.11	0	55	-155.03	0.09	205	105	-128.53	0.19

**A6QNL0**	120	85	-97.25	0.17	130	100	-226.74	0.20	80	120	-141.32	0.14
**F1MJK3**	40	120	-110.04	0.10	40	120	-257.67	0.21	85	15	-121.67	0.15
**A4IFG0**	320	55	-105.24	0.20	75	120	-215.30	0.24	195	145	-203.21	0.16
**A7YWM2**	260	85	-83.56	0.06	260	85	-207.52	0.13	310	85	-125.63	0.03
**P62998**	70	50	-86.56	0.17	75	55	-138.79	0.02	235	55	-141.42	0.15
**P13753**	55	50	-116.17	0.15	60	50	-230.60	0.25	200	40	-155.41	0.17
**F1MZQ5**	50	40	-130.02	0.15	55	40	-252.11	0.25	55	50	-174.99	0.12
**P13752**	200	40	-110.72	0.20	210	50	-205.93	0.19	55	40	-178.38	0.10
**Q3SZB7**	135	145	-83.03	0.12	260	95	-204.75	0.19	180	105	-127.57	0.11
**F1N6G4**	195	145	-126.74	0.20	200	145	-243.25	0.31	80	140	-126.91	0.18
**Q2TBL2**	315	125	-61.97	0.04	320	120	-164.71	0.09	40	120	-170.68	0.04
**A1L5C6**	235	55	-89.24	0.18	160	45	-182.05	0.24	120	85	-188.26	0.12
**Q3ZCK3**	270	40	-81.62	0.21	250	95	-201.69	0.26	160	85	-168.99	0.15
**A6QPP2**	330	120	-104.52	0.10	260	95	-187.04	0.24	65	95	-153.86	0.07
**Q3T0Q4**	220	65	-105.39	0.19	225	65	-189.12	0.32	105	60	-127.22	0.18
**Q58DK5**	185	100	-87.19	0.19	130	80	-187.13	0.25	15	125	-145.64	0.15
**A3KLR9**	145	165	-112.07	0.15	145	165	-209.01	0.22	220	80	-134.99	0.06
**F1MH40**	145	55	-84.14	0.18	85	75	-175.52	0.25	150	150	-188.90	0.02
**F1MLU7**	200	70	-70.52	0.16	25	135	-180.53	0.06	335	155	-152.77	0.17
**P63103**	115	150	-68.13	0.21	110	150	-163.87	0.35	50	115	-138.54	0.11
**G3X8C8**	35	145	-101.32	0.17	35	145	-235.43	0.28	355	115	-146.51	0.06
**Q3SZR3**	190	105	-78.89	0.15	190	105	-183.64	0.28	70	100	-128.82	0.07
**Q95121**	85	145	-127.10	0.18	55	95	-235.75	0.20	195	95	-169.94	0.11
**F1MXG6**	115	110	-68.28	0.04	120	115	-185.60	0.05	170	120	-170.90	0.05
**G5E5T5**	265	155	-81.12	0.02	130	30	-155.23	0.01	190	125	-159.92	0.11
**P08166**	330	125	-94.21	0.23	195	45	-173.10	0.27	125	25	-143.55	0.01
**A3KN27**	175	100	-101.47	0.07	210	145	-225.18	0.09	185	15	-145.08	0.19
**Q3SX46**	205	10	-98.88	0.20	215	60	-206.31	0.26	285	140	-167.23	0.17
**A2VE47**	285	140	-106.67	0.20	285	145	-198.43	0.28	225	15	-160.35	0.01
**F1N0Y0**	110	45	-113.20	0.11	110	50	-222.13	0.25	35	150	-160.48	0.12
**F1MR22**	245	95	-79.26	0.19	265	20	-185.78	0.05	90	140	-177.96	0.16
**A3KMY1**	5	135	-113.21	0.03	20	60	-213.93	0.20	115	110	-141.52	0.04
**Q8WML4**	295	55	-67.19	0.08	70	100	-123.90	0.15	195	105	-121.41	0.16
**A5D9E9**	165	120	-107.54	0.12	165	120	-293.74	0.18	45	95	-115.82	0.08
**E1BFG0**	300	125	-115.39	0.18	210	90	-216.02	0.26	80	70	-125.03	0.13
**E1BFG1**	355	115	-104.67	0.04	355	115	-215.54	0.14	110	150	-147.84	0.18
**Q17QK3**	70	150	-106.04	0.07	80	145	-233.21	0.12	145	45	-142.00	0.17
**E1B9D7**	105	70	-62.86	0.01	95	85	-199.71	0.13	210	135	-174.21	0.13
**Q3SYW2**	0	65	-119.31	0.10	200	85	-220.83	0.26	25	105	-169.33	0.12
**G3MXG6**	45	55	-72.40	0.07	140	30	-165.22	0.25	10	125	-123.36	0.05
**G3N3R5**	20	60	-108.60	0.08	10	85	-243.78	0.04	90	135	-163.31	0.10
**F1MSQ6**	310	60	-127.52	0.16	310	60	-219.12	0.26	350	120	-173.97	0.11
**P37980**	40	55	-86.55	0.01	165	115	-183.24	0.29	105	80	-171.70	0.11
**F1MMD7**	135	35	-84.74	0.14	305	80	-190.30	0.23	225	40	-139.40	0.16
**Q5E9G7**	335	50	-41.64	0.11	65	70	-92.79	0.12	65	70	-80.03	0.05
**F1MIH9**	275	75	-135.92	0.20	130	100	-241.98	0.21	50	0	-196.13	0.10

**F1N1Z6**	15	130	-128.50	0.16	55	0	-248.83	0.23	275	75	-180.04	0.17
**F1MMR8**	25	105	-116.50	0.15	25	105	-207.95	0.30	120	135	-157.19	0.11
**P68138**	10	125	-79.99	0.09	10	115	-192.43	0.13	315	40	-107.02	0.16
**G3X6K8**	125	150	-99.80	0.09	345	50	-157.37	0.22	310	60	-178.62	0.14
**A4IFP2**	165	115	-122.92	0.12	25	30	-220.26	0.11	170	105	-171.53	0.09
**F1N076**	290	85	-105.43	0.15	215	105	-244.14	0.24	100	80	-111.20	0.03
**P00978**	355	120	-129.04	0.19	45	90	-220.64	0.25	200	85	-156.47	0.13
**Q32L50**	135	75	-104.25	0.04	100	40	-217.98	0.03	75	145	-176.22	0.09
**Q5DPW9**	140	145	-87.78	0.21	150	135	-141.56	0.33	20	35	-155.51	0.04
**A4IFS7**	95	50	-88.47	0.17	10	75	-174.35	0.29	240	140	-150.19	0.06
**P40682**	115	25	-69.72	0.14	315	70	-159.45	0.12	295	90	-141.72	0.04
**P63048**	10	100	-103.86	0.21	55	160	-185.19	0.31	15	65	-139.23	0.12
**Q2TBX4**	205	120	-76.33	0.18	355	50	-190.38	0.24	230	120	-142.93	0.03
**G3X7W8**	325	15	-97.69	0.06	340	15	-192.58	0.17	195	55	-145.71	0.04
**F1MT12**	110	45	-76.05	0.19	260	70	-175.66	0.20	270	150	-95.70	0.10
**F1MI98**	35	80	-84.15	0.11	30	75	-281.89	0.10	120	100	-172.98	0.01
**A7YY28**	210	105	-97.82	0.19	60	130	-179.28	0.27	330	50	-166.55	0.19
**F1N6H6**	30	150	-79.67	0.13	195	50	-182.09	0.02	235	90	-181.71	0.15
**F1MJV6**	235	115	-110.58	0.04	165	25	-219.62	0.05	350	115	-146.85	0.15
**Q27960**	260	150	-81.20	0.19	10	120	-165.69	0.23	265	70	-143.14	0.11
**Q9N0V4**	330	45	-113.30	0.22	210	65	-207.66	0.23	245	65	-152.56	0.15
**E1BBP1**	235	90	-123.54	0.18	340	170	-246.77	0.23	115	110	-144.46	0.03
**F1N3M3**	270	145	-57.79	0.14	265	140	-109.49	0.22	35	80	-163.82	0.11
**G3MYM8**	115	95	-119.46	0.03	295	25	-264.19	0.05	95	45	-143.07	0.19
**A7YWK3**	185	110	-87.56	0.05	185	110	-261.26	0.09	220	65	-86.22	0.17
**P02666**	15	65	-99.90	0.18	210	85	-123.25	0.39	150	140	-126.42	0.17
**Q58D62**	175	125	-105.41	0.08	65	55	-209.55	0.22	355	25	-147.89	0.17
**Q3SZ62**	320	40	-102.25	0.20	5	55	-199.10	0.24	45	155	-158.48	0.17
**A6QNV3**	175	40	-57.80	0.18	175	40	-82.16	0.36	270	40	-194.55	0.08
**F1MJQ1**	90	155	-80.78	0.22	100	115	-168.16	0.29	175	115	-123.84	0.18
**Q28065**	130	100	-59.32	0.20	235	120	-172.00	0.09	270	110	-178.47	0.14
**G3MXC4**	180	45	-52.54	0.21	205	25	-88.84	0.00	235	25	-126.55	0.19
**G8JKV7**	10	10	-94.16	0.20	340	0	-183.21	0.25	250	120	-265.27	0.12
**Q3MHM5**	130	135	-95.55	0.19	130	135	-188.45	0.22	145	95	-150.53	0.14
**P80195**	20	90	-76.06	0.21	355	110	-170.46	0.31	55	75	-195.78	0.06
**P24627**	205	80	-163.89	0.18	200	80	-241.94	0.21	325	80	-126.16	0.17
**G3MYU2**	225	150	-102.67	0.14	210	145	-240.21	0.15	130	145	-228.98	0.08
**F1N529**	110	115	-89.83	0.15	10	70	-184.13	0.23	250	85	-168.80	0.14
**Q05443**	240	100	-90.02	0.17	240	100	-207.51	0.29	275	120	-129.73	0.12
**Q5E9X0**	105	60	-111.25	0.01	140	70	-194.90	0.30	245	70	-200.53	0.10
**Q2T9S4**	60	20	-112.28	0.19	335	140	-160.35	0.26	140	70	-150.25	0.16
**P00570**	245	130	-82.70	0.20	185	150	-166.07	0.29	240	100	-179.04	0.13
**Q0IIH5**	15	100	-65.60	0.02	355	105	-154.39	0.02	160	100	-228.42	0.08
**Q148C9**	70	75	-80.96	0.22	110	70	-193.43	0.29	265	70	-134.87	0.18
**A6QLY7**	160	100	-149.09	0.07	160	100	-265.12	0.19	55	20	-152.07	0.16
**Q1RMJ6**	145	95	-93.70	0.17	145	95	-198.54	0.26	10	100	-144.84	0.06

**A5PJH7**	55	75	-152.92	0.08	55	75	-202.33	0.23	110	70	-148.21	0.11
**F1MMI6**	250	120	-155.10	0.13	250	115	-280.72	0.09	235	120	-109.00	0.05
**G3N2D8**	245	90	-110.29	0.19	295	35	-229.72	0.22	180	45	-77.34	0.20
**Q4U5R3**	275	120	-87.22	0.16	240	65	-185.84	0.18	205	95	-160.41	0.14
**P15497**	250	65	-128.45	0.12	250	65	-327.49	0.20	265	20	-158.99	0.17
**G3X745**	125	145	-156.01	0.04	130	140	-268.81	0.15	175	40	-87.92	0.16
**P63258**	160	65	-85.57	0.19	340	100	-182.75	0.18	80	55	-120.46	0.09
**G3N0T0**	270	110	-94.51	0.18	275	110	-204.84	0.26	115	115	-145.20	0.10
**Q3T000**	355	150	-77.64	0.22	340	150	-153.97	0.28	215	150	-183.41	0.07
**B0JYR0**	270	40	-149.84	0.13	270	40	-235.60	0.27	205	80	-232.30	0.15
**P13696**	205	110	-85.13	0.19	210	25	-181.69	0.30	10	90	-125.53	0.19
**P56652**	55	65	-120.98	0.08	105	135	-208.67	0.18	265	70	-153.35	0.18
**F1MW38**	275	130	-71.90	0.10	275	130	-153.64	0.24	250	80	-154.92	0.08
**M0QVY0**	330	50	-77.39	0.06	355	75	-201.80	0.09	255	130	-121.65	0.16
**Q2KI84**	70	100	-86.16	0.17	65	95	-173.80	0.22	20	120	-143.11	0.12
**F1MEP6**	300	130	-95.82	0.17	15	70	-145.76	0.26	70	65	-191.27	0.07
**O97817**	330	135	-104.54	0.19	30	100	-244.30	0.13	125	135	-160.26	0.13
**E1BGJ4**	160	110	-140.30	0.09	170	115	-297.83	0.23	160	140	-207.25	0.01
**Q0II48**	190	165	-111.29	0.20	190	160	-218.55	0.29	290	40	-165.62	0.08
**F1N2D5**	255	50	-97.76	0.19	235	150	-204.92	0.19	335	165	-133.36	0.17
**P21793**	10	130	-111.74	0.18	40	115	-206.44	0.23	15	125	-150.77	0.06
**F1MFI4**	10	125	-114.42	0.17	160	110	-258.98	0.09	10	95	-168.14	0.16
**Q8SPP7**	340	165	-100.48	0.20	350	170	-202.45	0.22	170	105	-146.18	0.08
**F1MCL0**	20	125	-148.96	0.10	20	125	-230.54	0.21	130	90	-211.81	0.08
**P46193**	180	60	-87.00	0.19	145	55	-193.78	0.14	190	60	-152.25	0.17
**F1MYX5**	170	105	-95.65	0.14	5	35	-225.75	0.28	295	125	-198.22	0.16
**F1MM13**	130	90	-120.17	0.11	130	90	-241.40	0.22	300	130	-140.97	0.14
**G5E6M1**	120	135	-111.01	0.09	200	75	-190.25	0.05	100	85	-131.47	0.13
**Q29437**	70	65	-123.44	0.11	70	65	-278.36	0.21	295	10	-172.99	0.12
**Q2TBX9**	20	120	-90.98	0.14	20	120	-235.38	0.21	285	95	-144.19	0.04
**F1MYJ3**	105	40	-105.81	0.21	295	40	-279.83	0.22	125	115	-139.76	0.10
**Q2HJB6**	180	145	-118.70	0.13	160	140	-229.48	0.20	195	165	-166.52	0.18
**Q08DL0**	250	80	-109.23	0.11	185	105	-188.67	0.22	190	95	-147.17	0.16
**Q2KIX7**	10	145	-110.30	0.21	215	75	-171.37	0.25	30	100	-153.66	0.05
**E1B6Z6**	175	130	-80.05	0.24	260	130	-151.13	0.30	170	115	-240.68	0.07
**Q1RMU6**	150	170	-113.17	0.16	65	15	-191.73	0.27	120	85	-142.24	0.09
**F1MMV6**	205	100	-103.41	0.11	150	35	-168.48	0.29	265	115	-182.59	0.15
**E1BD43**	210	90	-116.89	0.18	210	95	-231.65	0.24	110	110	-140.53	0.16
**Q32KV6**	260	125	-86.35	0.05	290	155	-185.16	0.16	270	80	-168.20	0.11
**Q2TA49**	235	125	-114.93	0.01	235	125	-159.97	0.23	25	60	-173.08	0.08
**Q9GLE5**	190	80	-99.20	0.11	205	155	-209.58	0.23	340	95	-229.36	0.05
**A5PKH3**	130	145	-113.50	0.18	130	140	-179.91	0.29	80	130	-154.98	0.13
**P69678**	170	55	-125.15	0.17	165	50	-168.98	0.27	105	85	-194.35	0.06
**F1MDS0**	40	80	-93.72	0.06	40	80	-114.67	0.24	205	65	-143.60	0.18
**F1MRQ8**	215	115	-107.25	0.20	215	115	-240.20	0.29	35	75	-111.00	0.16
**Q2TBI0**	15	160	-89.01	0.17	255	115	-201.36	0.16	235	125	-158.59	0.02

**Q0V8R6**	55	55	-114.55	0.09	225	105	-182.51	0.17	205	160	-161.25	0.15
**E1B748**	340	90	-139.75	0.14	350	110	-274.08	0.08	300	30	-125.98	0.18
**F1MEW1**	25	60	-132.53	0.11	25	60	-211.47	0.18	55	60	-153.95	0.15
**G3X701**	340	175	-73.29	0.14	310	170	-162.31	0.24	215	115	-186.56	0.14
**Q32LG3**	205	70	-92.68	0.22	60	20	-210.16	0.27	270	110	-142.57	0.07
**F1MTI7**	80	125	-99.71	0.19	80	60	-172.46	0.25	170	55	-148.13	0.14
**Q3ZCJ8**	75	55	-111.48	0.19	240	140	-276.82	0.17	45	80	-118.69	0.07
**Q3SZ18**	265	115	-91.93	0.18	265	115	-210.04	0.29	130	145	-167.05	0.17
**F1MB08**	120	85	-94.67	0.13	120	85	-217.86	0.20	345	65	-145.12	0.10
**Q28161**	265	75	-120.36	0.06	270	80	-211.27	0.25	210	90	-184.91	0.15
**P0CH28**	110	110	-89.16	0.18	105	105	-160.26	0.30	70	15	-169.93	0.13
**E1BGX8**	130	130	-140.28	0.01	40	85	-221.01	0.28	130	60	-145.04	0.13
**P02081**	220	105	-89.99	0.20	40	85	-152.02	0.25	300	90	-147.50	0.18
**E1BKQ9**	115	55	-82.38	0.07	150	45	-147.85	0.09	195	95	-107.94	0.18
**F1MD95**	15	130	-51.04	0.06	30	165	-75.58	0.03	55	100	-202.27	0.13
**Q3T0E5**	205	150	-105.78	0.21	160	170	-175.21	0.22	285	75	-105.28	0.08
**A6QL77**	215	115	-101.68	0.17	220	115	-249.07	0.29	140	105	-149.34	0.12
**P68265**	190	115	-80.45	0.18	205	50	-226.10	0.24	60	20	-120.88	0.03
**Q9TUM6**	335	85	-82.57	0.12	175	90	-196.37	0.12	260	65	-134.77	0.18
**Q3ZBV9**	160	130	-122.00	0.21	230	45	-203.77	0.28	50	25	-167.35	0.11
**F1MVS9**	150	115	-109.70	0.07	280	40	-212.47	0.08	165	135	-152.49	0.15
**F1MJM4**	150	80	-98.57	0.17	155	80	-192.49	0.29	250	50	-131.14	0.09
**Q3SZV7**	165	135	-119.86	0.17	315	130	-189.48	0.25	155	80	-149.08	0.13
**F1N045**	0	40	-65.60	0.11	0	40	-156.65	0.17	290	50	-159.02	0.17
**P80311**	260	60	-96.13	0.22	185	95	-154.35	0.27	205	115	-156.67	0.16
**F1N362**	45	25	-94.14	0.05	15	145	-220.87	0.06	205	150	-152.92	0.18
**Q8MI01**	20	95	-64.56	0.02	275	100	-124.74	0.09	15	130	-83.97	0.10
**Q2KJE7**	85	120	-144.25	0.11	55	100	-250.28	0.26	220	115	-198.59	0.12
**F1MXX6**	300	90	-98.18	0.21	325	80	-183.01	0.25	110	55	-118.55	0.05
**Q3MHJ4**	315	55	-61.47	0.21	45	135	-141.58	0.24	215	110	-141.60	0.05
**A5PKB4**	15	15	-89.69	0.20	125	60	-181.52	0.26	125	135	-194.10	0.04
**P02769**	140	105	-83.31	0.17	140	105	-187.85	0.26	160	130	-168.75	0.17
**A5D7Q6**	285	75	-64.73	0.15	285	75	-113.73	0.27	10	60	-136.52	0.06
**Q3SYS6**	160	160	-88.26	0.20	155	160	-186.90	0.28	85	120	-182.44	0.12
**P38657**	65	45	-95.50	0.11	35	140	-243.40	0.09	140	65	-135.31	0.17
**P00711**	20	80	-48.10	0.21	0	90	-149.41	0.31	340	80	-128.03	0.17
**A6QL48**	340	50	-126.39	0.18	340	50	-179.54	0.35	145	155	-163.09	0.17
**E1BI98**	285	10	-124.20	0.11	320	10	-268.57	0.15	130	95	-184.36	0.11
**E1BM57**	195	100	-79.34	0.19	190	105	-182.50	0.33	180	30	-119.74	0.03
**Q6ZXD2**	295	90	-110.21	0.20	290	95	-211.25	0.22	125	105	-113.97	0.17
**A5PK77**	115	125	-82.37	0.22	115	125	-204.68	0.23	265	90	-138.88	0.12
**G3MZ71**	220	90	-83.48	0.08	220	95	-201.78	0.20	90	70	-152.96	0.17
**A6QPQ2**	0	0	-0.00	2.00	0	0	-0.00	2.00	320	85	-127.92	0.07
**F1MU22**	275	125	-115.89	0.18	280	120	-256.38	0.27	180	25	-162.08	0.16
**Q1RMN9**	20	135	-95.99	0.15	355	130	-200.40	0.17	125	100	-191.39	0.05
**F1MGN0**	235	170	-156.69	0.18	245	175	-272.67	0.22	25	110	-104.86	0.08

O02659	140	25	-124.48	0.07	105	60	-226.80	0.15	325	125	-135.14	0.06
P79121	270	85	-78.57	0.06	105	140	-159.94	0.27	155	20	-177.31	0.09
F1MLI8	40	110	-64.45	0.11	250	90	-159.61	0.05	0	0	-0.00	2.00
O02751	90	70	-91.16	0.19	10	15	-177.83	0.04	185	40	-137.54	0.08
F1N3Q7	220	110	-73.48	0.09	205	110	-205.64	0.08	115	125	-175.11	0.04
Q3ZBG1	200	135	-80.58	0.20	105	125	-153.42	0.24	295	90	-175.42	0.15
A5D7R9	70	125	-77.46	0.21	265	90	-192.43	0.26	225	150	-207.56	0.14
E1B9M9	345	140	-78.87	0.22	140	65	-174.16	0.30	165	80	-151.35	0.14
Q2KIV9	140	160	-103.17	0.19	125	155	-174.24	0.18	250	130	-186.44	0.06
A7E3Q8	130	95	-123.48	0.15	130	95	-244.43	0.25	45	130	-144.77	0.08
Q5E984	335	75	-87.23	0.20	340	80	-149.55	0.38	140	165	-133.87	0.15
E1BLC2	25	140	-83.74	0.21	180	40	-170.90	0.29	190	105	-158.94	0.14
Q2KIV8	110	130	-86.33	0.17	80	115	-235.99	0.25	340	50	-170.94	0.16
A8YXZ2	125	100	-172.69	0.08	220	130	-189.90	0.27	290	10	-221.94	0.13
Q3T0K1	180	30	-84.24	0.18	225	125	-199.43	0.23	5	90	-127.74	0.18
Q3ZBW5	150	45	-83.02	0.19	140	40	-158.12	0.28	175	145	-134.99	0.16
F1MNI4	260	60	-85.86	0.17	130	115	-163.36	0.31	70	135	-153.09	0.09
P16368	340	125	-65.28	0.19	85	160	-156.17	0.23	325	45	-129.58	0.15
A1A4K5	195	140	-138.41	0.02	295	100	-227.59	0.12	280	40	-168.07	0.17
Q9TTE1	340	90	-84.26	0.17	285	95	-197.36	0.21	235	5	-134.45	0.16
G3MXH2	115	95	-122.75	0.21	305	115	-227.80	0.26	55	25	-125.93	0.17
E1BI28	220	110	-85.71	0.19	250	155	-175.52	0.28	50	40	-145.32	0.03
A6QLR1	60	115	-50.62	0.06	205	130	-113.78	0.00	35	60	-169.36	0.17
F1MTT3	130	130	-83.02	0.13	75	85	-151.67	0.22	70	50	-136.07	0.09
A7E3W2	345	140	-105.64	0.17	340	140	-209.22	0.12	175	125	-183.89	0.09
F1N1N6	330	90	-121.82	0.06	315	100	-210.68	0.11	90	55	-108.14	0.18
G3MYW7	80	95	-78.71	0.14	185	135	-214.56	0.25	200	160	-154.02	0.16
F1N3H1	55	50	-85.58	0.18	30	120	-153.92	0.28	100	110	-144.76	0.02
Q08E20	165	120	-129.82	0.17	180	125	-228.45	0.27	210	65	-119.12	0.06
A6QP32	5	70	-69.82	0.12	70	45	-170.80	0.11	110	70	-96.37	0.06
F1N4Y5	40	55	-107.60	0.16	45	60	-245.62	0.25	245	150	-134.49	0.16
F1MIQ2	95	110	-98.75	0.03	335	100	-191.29	0.09	185	135	-151.08	0.10
F1MKH8	220	60	-101.53	0.12	325	30	-199.87	0.29	175	20	-147.19	0.15
Q08DV2	245	110	-62.35	0.20	25	115	-161.23	0.26	325	90	-160.02	0.10
Q28193	65	25	-104.56	0.14	70	135	-202.75	0.25	345	140	-147.34	0.13
G1K122	180	145	-91.01	0.18	230	125	-190.43	0.17	150	45	-127.03	0.16
P08037	85	30	-103.27	0.20	235	70	-186.25	0.22	115	95	-169.00	0.18
Q6B856	320	70	-95.19	0.17	260	15	-196.00	0.27	235	125	-145.94	0.15
E1BC58	45	30	-76.53	0.21	65	25	-161.79	0.27	190	140	-200.88	0.09
Q0VCX1	305	55	-105.18	0.20	280	40	-203.27	0.33	270	75	-121.55	0.18
Q3T0I2	235	20	-84.17	0.18	230	20	-184.35	0.23	95	140	-118.28	0.18
Q3T052	20	85	-93.65	0.19	240	105	-236.77	0.13	150	80	-153.37	0.14
Q3ZC09	135	150	-132.13	0.19	130	130	-278.98	0.27	140	20	-185.81	0.06
Q5E9F7	120	105	-73.86	0.22	15	80	-148.50	0.27	180	80	-143.48	0.14
A6H7J6	220	40	-82.67	0.07	300	95	-203.11	0.16	210	100	-177.35	0.02
Q17QG8	205	120	-84.15	0.04	200	115	-176.50	0.21	90	45	-156.58	0.14

P02702	110	40	-150.84	0.20	110	40	-216.02	0.36	200	70	-112.59	0.09
Q9N2I2	115	130	-148.00	0.17	115	130	-218.05	0.32	5	100	-173.68	0.01
E1BKZ9	240	165	-135.90	0.18	40	105	-209.28	0.23	210	75	-146.00	0.03
P12763	235	130	-97.21	0.19	270	140	-157.69	0.15	125	145	-138.11	0.10
F1ME65	25	25	-83.29	0.03	345	150	-194.27	0.00	250	60	-176.00	0.14
A2I7N2	225	45	-75.86	0.16	115	125	-191.90	0.24	275	80	-178.58	0.03
A2I7N3	325	75	-75.08	0.16	115	125	-186.39	0.28	115	115	-141.69	0.12
F1MLW8	245	95	-64.33	0.18	255	90	-155.92	0.21	250	105	-178.09	0.18
Q3SZF2	290	25	-91.76	0.23	180	105	-186.91	0.30	120	125	-149.04	0.14
A3KMV5	90	110	-92.89	0.08	320	75	-180.26	0.16	305	70	-124.05	0.16
E1BAB5	5	100	-106.84	0.06	10	90	-281.46	0.04	40	10	-178.70	0.01
Q5EA79	90	50	-93.86	0.17	90	45	-197.64	0.30	35	95	-134.57	0.13
Q2LGB5	285	30	-75.99	0.12	160	65	-159.75	0.18	290	25	-132.49	0.18
A6QPT7	320	145	-82.42	0.12	340	65	-212.28	0.22	195	25	-106.53	0.07
Q2KJ51	215	90	-117.78	0.05	210	100	-212.02	0.12	110	40	-195.00	0.18
P54228	145	100	-100.02	0.21	155	80	-196.25	0.27	110	125	-228.59	0.06
F1MDD8	140	20	-106.17	0.03	105	25	-242.43	0.12	205	120	-142.67	0.06
Q1JPE3	250	60	-134.26	0.19	15	5	-217.04	0.23	235	130	-141.60	0.16
F1MG91	155	55	-107.45	0.16	305	0	-217.28	0.21	50	100	-193.58	0.15
Q2KIW4	285	145	-126.45	0.18	275	80	-205.64	0.19	130	130	-221.94	0.15
Q17QC1	85	70	-106.82	0.20	255	110	-217.36	0.27	140	55	-168.10	0.10
E1BJH7	50	35	-81.03	0.21	245	125	-184.61	0.23	220	40	-133.55	0.10
A5D7S4	55	85	-99.11	0.15	295	60	-205.76	0.19	120	105	-108.22	0.18
A5PKC2	50	45	-93.00	0.12	220	30	-167.58	0.23	340	170	-146.42	0.04
Q2KJ64	190	115	-112.53	0.22	5	80	-219.24	0.22	160	65	-156.48	0.10
Q9XSC6	130	110	-86.55	0.17	95	80	-240.38	0.08	325	90	-198.56	0.04
Q58DS9	145	115	-79.38	0.20	150	120	-161.55	0.28	240	65	-169.65	0.08
Q2KJD0	325	70	-104.63	0.19	325	75	-211.62	0.11	335	130	-172.47	0.07
Q2KIT0	295	90	-119.63	0.18	300	85	-191.24	0.26	220	105	-142.92	0.17
F1MMK9	165	105	-124.32	0.13	170	100	-240.67	0.13	160	75	-148.27	0.09
F1MUP9	335	120	-137.27	0.16	335	115	-210.70	0.31	200	130	-153.03	0.03
F1N1C7	245	115	-132.69	0.02	240	120	-292.58	0.14	290	80	-182.37	0.07
E1BJB1	215	15	-109.63	0.16	245	5	-196.56	0.27	315	30	-181.16	0.12
Q3ZBS7	60	90	-97.98	0.14	60	90	-224.05	0.25	60	90	-185.38	0.12
Q3SYU6	310	30	-117.15	0.19	280	95	-227.28	0.20	335	120	-162.17	0.15
Q6R8F2	70	65	-47.20	0.10	115	100	-82.36	0.01	240	120	-219.84	0.03
Q3SX14	260	90	-95.04	0.10	330	125	-234.87	0.10	220	10	-176.99	0.16
A7MBI6	325	95	-92.66	0.19	160	85	-181.18	0.29	35	75	-141.02	0.08
P02676	95	120	-113.01	0.04	150	35	-215.44	0.05	160	40	-144.61	0.06
A6QP39	30	75	-91.29	0.07	35	75	-226.25	0.14	160	85	-136.34	0.11
Q2NKZ9	340	115	-99.96	0.19	205	25	-190.26	0.29	10	125	-65.92	0.05
Q148H5	195	130	-96.43	0.02	215	145	-242.30	0.12	80	75	-147.84	0.13
O46406	70	65	-106.71	0.09	270	70	-214.38	0.20	230	10	-166.48	0.16
P19120	70	40	-92.07	0.16	160	75	-205.44	0.19	275	70	-128.86	0.14
P09487	125	20	-80.54	0.16	5	155	-169.47	0.28	130	110	-150.29	0.14
Q0VCU1	175	145	-96.92	0.16	130	105	-221.13	0.23	190	115	-173.22	0.17

**P0CB32**	335	130	-105.37	0.04	350	25	-250.85	0.16	210	35	-138.98	0.16
**P81187**	245	70	-133.14	0.11	205	80	-217.05	0.23	285	95	-156.05	0.05
**Q5E9E3**	320	90	-118.62	0.17	325	90	-245.41	0.25	165	105	-225.58	0.09
**P26779**	125	85	-83.39	0.15	145	130	-199.74	0.24	285	95	-142.32	0.16
**P98140**	125	115	-96.74	0.12	125	130	-201.55	0.03	270	155	-153.10	0.12
**A7Z014**	335	90	-99.59	0.21	285	135	-210.71	0.18	205	75	-204.40	0.14
**Q58DP6**	295	85	-110.94	0.09	295	85	-181.47	0.27	315	15	-146.53	0.19
**Q3T083**	140	165	-69.66	0.20	140	40	-156.90	0.28	70	95	-164.97	0.11
**A6H6X2**	25	70	-125.48	0.21	275	160	-206.30	0.23	165	105	-160.78	0.09
**Q08E14**	160	35	-127.23	0.12	310	30	-304.34	0.02	45	20	-199.31	0.14
**P79136**	335	150	-86.64	0.22	345	150	-191.12	0.21	105	105	-138.56	0.16
**Q03247**	345	80	-95.99	0.23	240	95	-150.76	0.29	80	105	-131.54	0.07
**P35445**	250	105	-124.08	0.19	280	105	-249.97	0.13	50	55	-135.72	0.06
**Q769I5**	135	135	-113.75	0.02	135	135	-231.96	0.06	130	80	-164.30	0.17
**P04272**	230	100	-118.60	0.18	225	105	-257.19	0.27	350	135	-138.94	0.17
**G3MXB5**	125	35	-67.91	0.17	120	10	-150.93	0.14	135	110	-111.47	0.16
**G8JKW7**	310	30	-75.86	0.17	110	125	-186.02	0.25	80	100	-124.56	0.16
**P17453**	260	120	-107.15	0.10	270	115	-216.63	0.16	150	55	-107.47	0.15
**A4IFN4**	205	145	-101.98	0.05	200	135	-227.64	0.06	240	125	-174.72	0.16
**Q17QG2**	150	125	-114.36	0.17	150	125	-274.11	0.26	150	125	-180.85	0.14
**Q2KIS7**	165	115	-72.23	0.07	160	105	-192.60	0.18	155	105	-119.69	0.11
**F1MU34**	195	130	-136.06	0.15	55	60	-230.28	0.18	270	115	-184.03	0.05
**F1MGY9**	185	15	-108.37	0.19	300	35	-178.25	0.28	200	135	-170.01	0.03
**A5PJ69**	270	155	-108.81	0.13	265	30	-192.36	0.22	345	80	-139.57	0.19
**Q3SYR0**	285	85	-94.55	0.18	290	100	-161.14	0.26	340	150	-161.57	0.12
**Q0V7N2**	25	55	-146.09	0.18	30	45	-257.73	0.13	250	105	-181.47	0.16
**E1BKS1**	350	135	-80.31	0.21	345	135	-173.69	0.33	135	135	-201.77	0.01
**F1N430**	15	110	-61.35	0.16	95	30	-164.17	0.21	340	45	-184.78	0.09
**P50291**	50	55	-91.28	0.09	185	165	-198.36	0.23	280	155	-159.01	0.02
**G3MXK6**	165	160	-97.82	0.20	250	135	-208.56	0.19	235	100	-178.02	0.16
**F1N431**	45	15	-111.58	0.17	50	20	-250.92	0.27	125	125	-143.27	0.14
**Q58D04**	235	55	-80.32	0.14	240	50	-179.17	0.28	145	130	-148.06	0.13
**G3N0Q8**	125	75	-110.80	0.22	35	145	-197.37	0.23	325	70	-124.17	0.12
**Q3SZB0**	70	95	-108.41	0.16	70	95	-198.51	0.27	295	85	-176.04	0.05
**A6QR28**	160	105	-92.38	0.15	165	100	-207.05	0.24	160	130	-142.89	0.04
**A6QLS9**	140	170	-94.99	0.17	220	170	-171.51	0.34	250	140	-172.87	0.05
**Q2TBX6**	175	160	-93.67	0.20	165	160	-203.11	0.25	5	45	-176.56	0.05
**Q3T0L2**	95	70	-80.30	0.18	165	50	-178.04	0.25	205	50	-153.26	0.17
**A5D7J6**	20	80	-80.95	0.16	25	90	-221.28	0.03	295	35	-134.89	0.18
**F1MU18**	10	110	-103.46	0.11	40	160	-221.71	0.07	60	10	-95.89	0.01
**E1BFQ6**	350	25	-99.84	0.19	115	40	-200.71	0.17	300	35	-136.70	0.19
**F1MQF0**	25	110	-89.47	0.14	75	100	-133.03	0.10	90	120	-134.57	0.04
**P61223**	305	65	-93.29	0.20	330	35	-180.73	0.26	150	20	-210.40	0.03
**Q3MHX6**	310	65	-120.24	0.14	250	125	-230.91	0.23	300	55	-157.93	0.07
**F1MXJ5**	310	160	-31.63	0.02	275	155	-93.69	0.11	90	95	-165.57	0.17
**P79334**	20	110	-116.97	0.09	70	85	-249.76	0.10	60	40	-168.15	0.19

**Q3T074**	330	115	-108.16	0.21	335	120	-176.80	0.33	250	25	-124.67	0.17
**F1MJ66**	290	55	-77.77	0.04	290	55	-210.68	0.11	250	130	-131.42	0.09
**E1BNY3**	225	40	-85.15	0.15	260	60	-181.71	0.01	280	100	-147.41	0.11
**Q2KIU3**	235	150	-109.31	0.19	55	35	-135.77	0.26	340	105	-123.20	0.18
**F1MYN5**	285	100	-95.01	0.12	280	100	-171.77	0.21	230	40	-157.75	0.08
**Q58CQ9**	210	95	-83.17	0.15	255	130	-188.55	0.27	260	160	-194.99	0.14
**P10790**	75	100	-53.13	0.22	255	25	-148.97	0.31	355	115	-152.33	0.15
**A5PJT7**	60	35	-117.16	0.21	300	25	-206.95	0.22	85	100	-118.48	0.17
**Q9BGI2**	265	40	-111.24	0.23	265	35	-174.52	0.33	315	40	-191.31	0.13
**Q9BGI3**	45	125	-87.64	0.18	130	25	-137.32	0.33	265	40	-172.21	0.19
**P06868**	15	125	-119.71	0.17	215	60	-190.41	0.17	335	155	-155.67	0.01
**P26201**	265	160	-103.32	0.15	255	155	-304.93	0.21	75	95	-143.51	0.10
**F1MR63**	350	115	-96.40	0.17	345	115	-179.53	0.11	115	35	-145.10	0.09
**P84081**	305	35	-88.61	0.22	85	40	-197.44	0.25	290	55	-189.72	0.03
**F1MNL4**	90	120	-86.44	0.00	275	100	-172.75	0.15	25	100	-182.48	0.07
**Q32PH8**	115	140	-114.79	0.16	155	15	-232.24	0.21	120	90	-82.68	0.00
**Q58DD4**	235	165	-57.48	0.00	230	165	-168.94	0.01	335	120	-137.60	0.11
**P84080**	295	35	-88.57	0.21	85	35	-217.29	0.25	310	65	-196.01	0.12
**F1N0T3**	205	50	-110.64	0.19	65	55	-215.94	0.24	165	50	-144.62	0.05
**A6QNX5**	355	35	-92.22	0.10	340	15	-252.99	0.13	155	170	-163.36	0.15
**F1N455**	245	135	-98.56	0.16	245	140	-279.93	0.20	235	170	-145.65	0.18
**Q2NKV1**	85	95	-116.80	0.20	230	125	-190.08	0.25	180	120	-153.51	0.11
**G3MYD9**	15	75	-86.12	0.02	20	75	-193.38	0.06	30	140	-129.96	0.05
**F2Z4D5**	340	80	-64.38	0.23	315	65	-187.14	0.25	275	55	-121.08	0.15
**E1BH94**	100	110	-108.02	0.15	105	40	-212.98	0.26	260	80	-88.36	0.02
**Q2HJ33**	310	120	-85.47	0.02	310	85	-195.74	0.11	120	80	-163.19	0.08
**A1L595**	185	115	-84.08	0.17	180	110	-236.41	0.13	135	40	-189.28	0.02
**Q0VC36**	220	15	-97.50	0.15	220	15	-185.07	0.31	60	60	-180.32	0.15
**Q58D84**	150	125	-93.25	0.19	200	95	-168.37	0.32	300	20	-108.84	0.07
**Q5QQ49**	295	125	-121.59	0.09	65	155	-208.46	0.10	300	130	-163.80	0.16
**F1MJQ3**	155	25	-104.32	0.19	165	85	-204.71	0.21	150	90	-174.78	0.11
**P25975**	90	40	-97.20	0.18	300	145	-190.81	0.26	225	150	-142.72	0.00
**Q1ZYR0**	305	105	-100.01	0.20	350	65	-234.62	0.28	115	70	-141.81	0.09
**Q0IIH7**	270	85	-113.05	0.20	105	85	-183.83	0.08	60	65	-127.18	0.12
**Q3SZ16**	295	80	-109.06	0.21	220	20	-239.48	0.14	230	20	-181.05	0.12
**Q2KJ93**	55	65	-91.71	0.15	60	65	-173.14	0.27	100	90	-167.08	0.08
**G5E6G2**	75	70	-82.21	0.22	310	45	-165.55	0.24	180	105	-169.32	0.07
**F1MEA1**	220	30	-105.47	0.08	150	25	-237.75	0.15	95	40	-159.34	0.18
**Q95116**	300	130	-125.48	0.19	295	125	-207.44	0.29	150	25	-182.40	0.07
**G3X7F3**	290	145	-55.40	0.15	290	140	-151.27	0.18	295	105	-141.78	0.00
**F1N036**	135	45	-93.68	0.10	135	40	-204.03	0.18	305	70	-155.70	0.08
**F1MX65**	60	65	-104.31	0.16	5	75	-208.34	0.03	315	60	-111.10	0.12
**E1BLC6**	25	125	-70.91	0.05	225	150	-162.19	0.04	60	155	-169.84	0.02
**F1MW08**	155	90	-122.31	0.15	260	50	-180.95	0.25	155	25	-165.39	0.16
**G3MWH1**	255	70	-45.23	0.01	85	85	-97.63	0.11	35	30	-150.53	0.00
**E1BHY6**	275	55	-92.72	0.18	130	120	-143.05	0.00	200	95	-149.41	0.12

**A6QLB0**	130	20	-112.21	0.06	285	25	-207.00	0.20	220	15	-169.43	0.13
**F1MY58**	185	55	-90.78	0.15	140	135	-183.37	0.23	185	110	-199.35	0.09
**F1N4X7**	315	70	-97.77	0.22	70	45	-204.60	0.26	310	85	-182.76	0.02
**Q2KHU0**	350	75	-105.84	0.24	225	90	-198.83	0.19	280	95	-172.63	0.10
**Q3T0A3**	175	145	-75.51	0.20	160	45	-151.52	0.29	280	160	-114.75	0.19
**A6QR46**	100	95	-65.88	0.22	90	140	-171.26	0.30	285	80	-201.32	0.10
**E1BEZ9**	140	140	-124.48	0.17	305	45	-191.40	0.21	260	100	-157.87	0.04
**F1MBS0**	245	80	-99.11	0.19	185	140	-176.95	0.21	295	60	-138.91	0.15
**Q865V6**	40	140	-110.48	0.11	45	145	-184.62	0.11	210	160	-198.42	0.12
**F1MX50**	25	165	-107.38	0.21	165	50	-225.72	0.13	255	25	-204.24	0.15
**E1BEL6**	340	50	-88.59	0.10	330	35	-171.15	0.16	220	100	-177.41	0.11
**E1BEL7**	200	150	-121.90	0.20	225	140	-181.54	0.29	5	5	-181.01	0.09
**P34955**	330	100	-85.61	0.20	95	135	-165.39	0.21	95	45	-162.15	0.03
**O77588**	5	10	-104.03	0.19	0	5	-218.69	0.29	290	55	-170.63	0.05
**A6QLZ0**	110	45	-126.68	0.21	5	75	-202.91	0.31	185	155	-156.16	0.18
**Q17QL7**	290	55	-89.20	0.18	285	55	-248.88	0.08	200	140	-137.27	0.15
**Q1RMR4**	130	175	-69.74	0.21	335	20	-155.88	0.27	240	80	-154.75	0.16
**Q1RMM9**	280	95	-103.33	0.13	275	100	-212.68	0.24	40	140	-157.01	0.09
**E1BJN3**	290	80	-116.37	0.05	210	95	-233.40	0.25	20	165	-162.80	0.19
**A6QQD5**	295	75	-108.74	0.20	320	85	-231.02	0.27	85	130	-144.04	0.08
**F1MU12**	185	110	-118.86	0.09	175	105	-226.56	0.02	180	60	-156.51	0.08
**Q3MHN2**	220	100	-114.70	0.12	210	110	-225.49	0.20	85	60	-158.92	0.01
**O18738**	75	70	-156.33	0.04	245	40	-302.49	0.15	210	95	-149.95	0.05
**O18739**	265	100	-96.71	0.02	260	105	-193.71	0.07	165	145	-129.37	0.06
**Q0P5L5**	230	105	-94.41	0.20	235	105	-186.36	0.30	90	140	-123.65	0.15
**A7MB70**	210	160	-143.17	0.14	220	90	-262.32	0.11	75	50	-168.68	0.12
**Q3SX32**	125	90	-92.31	0.03	125	85	-164.91	0.19	290	40	-176.31	0.14
**A7YWH4**	330	25	-83.42	0.18	335	25	-189.24	0.27	165	150	-97.11	0.02
**Q5BIR5**	90	115	-95.49	0.20	85	115	-244.10	0.27	175	70	-193.17	0.08
**F1MAV0**	265	10	-118.19	0.09	80	150	-216.33	0.06	250	95	-121.25	0.06
**A5D7R6**	245	100	-124.21	0.13	160	115	-202.92	0.25	280	150	-119.65	0.19
**Q1RMU3**	90	120	-93.07	0.08	115	30	-167.44	0.12	260	90	-124.80	0.14
**P21856**	320	110	-110.56	0.16	315	115	-234.42	0.25	75	80	-177.21	0.12
**P08728**	185	110	-108.94	0.14	185	110	-259.30	0.19	315	115	-163.05	0.11
**Q17QB3**	215	150	-130.76	0.18	220	145	-213.17	0.25	180	110	-186.07	0.09
**E1BIY5**	90	105	-111.56	0.10	90	80	-182.01	0.18	325	45	-190.73	0.04
**Q29444**	30	95	-138.46	0.15	25	95	-240.59	0.19	95	120	-138.05	0.13
**F1N4M7**	160	100	-112.95	0.17	160	100	-198.51	0.31	285	15	-166.61	0.19
**P10096**	280	10	-107.45	0.19	305	40	-170.23	0.28	160	100	-185.96	0.14
**P31976**	200	35	-109.05	0.14	300	100	-277.78	0.17	30	95	-180.56	0.14
**E1BMX0**	90	155	-71.67	0.20	260	90	-170.85	0.27	85	80	-158.35	0.04
**Q3T0F5**	300	130	-76.45	0.21	265	70	-166.44	0.24	125	90	-134.99	0.08
**F1MNT5**	265	100	-49.39	0.02	185	80	-125.33	0.24	265	35	-175.10	0.07
**Q8MJ16**	145	160	-62.95	0.10	20	155	-108.53	0.01	70	55	-160.08	0.05
**Q8MHZ9**	120	100	-128.25	0.17	115	140	-223.33	0.22	330	25	-126.90	0.17
**P02672**	315	135	-91.26	0.02	335	130	-203.95	0.19	335	75	-128.78	0.15

**P22444**	200	105	-101.61	0.17	40	55	-178.68	0.19	10	25	-136.77	0.12
**F1MNM2**	160	130	-123.34	0.16	315	60	-230.09	0.22	50	105	-158.45	0.06
**Q2TBK6**	245	70	-104.59	0.20	220	55	-159.47	0.28	75	125	-150.18	0.09
**G3MYZ3**	145	105	-84.18	0.16	140	105	-196.64	0.22	225	25	-168.32	0.01
**E1B9F6**	330	125	-103.49	0.23	255	40	-237.50	0.23	135	30	-164.46	0.07
**Q3SYU2**	200	75	-101.88	0.13	200	70	-200.71	0.21	200	40	-140.51	0.16
**Q3ZBZ1**	65	40	-131.39	0.17	75	35	-190.47	0.25	50	65	-158.91	0.00
**A6QPW7**	45	110	-147.42	0.16	115	65	-212.07	0.08	285	85	-140.02	0.12
**F1ML02**	200	80	-67.83	0.04	185	75	-134.16	0.21	270	125	-159.12	0.18
**A4FUZ0**	245	65	-97.22	0.13	145	85	-183.73	0.18	135	25	-214.15	0.11
**A6QPW6**	285	70	-72.15	0.16	260	75	-198.36	0.25	70	40	-174.91	0.08
**F1MSA1**	275	125	-140.13	0.20	95	80	-181.24	0.24	205	80	-95.80	0.06
**Q2KIF2**	320	40	-126.98	0.21	140	30	-301.95	0.21	245	65	-156.52	0.11
**E1BJ49**	285	85	-103.93	0.15	155	125	-215.61	0.13	260	75	-146.34	0.07
**F1MKS3**	45	100	-87.01	0.16	45	65	-183.41	0.22	45	110	-169.10	0.15
**P80025**	205	45	-98.89	0.18	5	165	-190.21	0.23	160	130	-171.86	0.13
**F1MZ63**	340	135	-103.42	0.18	65	135	-207.52	0.03	225	60	-153.15	0.12
**Q5EA01**	85	145	-120.49	0.21	100	120	-176.73	0.34	335	130	-139.27	0.11
**Q3MHP2**	340	20	-74.50	0.20	335	75	-140.94	0.32	250	70	-143.63	0.04
**Q5EAB4**	55	100	-104.90	0.16	45	105	-183.76	0.28	200	75	-161.54	0.13
**Q76LV2**	15	25	-76.73	0.12	225	120	-180.92	0.13	255	40	-161.14	0.11
**A5D7K0**	195	40	-100.34	0.17	215	45	-174.45	0.30	140	105	-135.35	0.14
**E1BNR9**	220	100	-130.19	0.16	215	100	-237.22	0.12	350	140	-209.03	0.14
**P01888**	80	15	-99.30	0.23	305	35	-153.27	0.27	150	80	-176.50	0.14
**P40673**	105	45	-107.36	0.14	105	45	-181.49	0.28	175	150	-108.78	0.01
**F1N780**	175	75	-77.39	0.05	60	160	-173.03	0.00	0	0	-0.00	2.00
**F1MLW7**	350	90	-75.42	0.19	235	30	-176.52	0.24	310	145	-127.79	0.15
**Q08E11**	35	105	-101.23	0.23	300	65	-151.21	0.28	155	60	-135.04	0.16
**A6QP90**	295	40	-77.28	0.04	280	50	-255.93	0.05	0	145	-137.97	0.15
**Q9TR36**	270	45	-84.55	0.22	290	15	-205.53	0.32	340	110	-154.74	0.15
**E1BIX9**	215	150	-112.19	0.13	205	145	-230.71	0.10	215	150	-171.10	0.11
**Q5E997**	70	50	-72.67	0.18	355	155	-174.29	0.22	280	20	-147.53	0.20
**P19660**	25	85	-183.54	0.02	15	95	-202.46	0.34	275	55	-154.04	0.02
**P01045**	150	100	-84.04	0.16	210	45	-159.99	0.25	170	55	-194.78	0.10
**P19661**	230	60	-161.36	0.22	170	50	-227.86	0.30	25	85	-218.29	0.00
**Q3SZ01**	270	115	-95.23	0.17	0	145	-185.71	0.22	120	30	-125.00	0.17
**P18341**	155	60	-95.87	0.18	240	65	-172.94	0.10	20	90	-127.96	0.16
**Q3ZC07**	100	165	-89.30	0.14	150	25	-196.30	0.13	85	35	-157.48	0.19
**Q28178**	280	30	-90.29	0.10	190	165	-226.87	0.18	250	125	-127.66	0.04
**F1MMS7**	255	105	-117.13	0.13	350	140	-251.16	0.30	220	100	-225.75	0.13
**A6QNW7**	0	0	-0.00	2.00	0	0	-0.00	2.00	80	15	-138.56	0.19
**F1ML75**	285	70	-72.69	0.11	70	100	-147.03	0.16	45	105	-144.29	0.18
**Q2KHZ8**	150	80	-125.31	0.19	155	80	-237.55	0.22	220	30	-111.14	0.15
**Q1JPB0**	100	115	-89.60	0.19	280	105	-172.14	0.25	150	155	-149.91	0.09
**Q0VD27**	210	165	-83.39	0.22	195	170	-186.84	0.24	55	30	-104.16	0.19
**Q29RI6**	180	60	-121.00	0.14	185	70	-210.25	0.27	85	85	-166.77	0.07

P56965	85	120	-95.76	0.18	355	90	-184.84	0.36	325	40	-139.36	0.16
O02853	105	60	-101.38	0.20	20	120	-153.35	0.27	240	10	-160.32	0.17
Q3ZCH5	325	60	-107.88	0.17	50	10	-236.44	0.24	55	175	-144.88	0.09
A6QR19	65	10	-138.29	0.15	50	5	-265.61	0.25	25	15	-135.79	0.13
E1BCJ2	280	60	-61.18	0.02	280	55	-188.38	0.03	125	60	-157.21	0.17
Q8SPJ1	280	55	-96.11	0.19	205	25	-195.81	0.24	260	55	-128.17	0.02
P01131	255	150	-74.20	0.19	120	135	-208.07	0.01	220	15	-146.50	0.16
F1N2W0	345	160	-79.90	0.18	75	135	-200.52	0.23	310	105	-211.30	0.11
P11151	35	70	-81.87	0.14	290	115	-172.47	0.22	70	60	-177.57	0.10
G5E589	305	35	-84.97	0.21	80	125	-215.80	0.21	200	140	-163.95	0.07
A4FV54	345	100	-76.73	0.20	340	100	-177.43	0.28	85	110	-166.88	0.04
E1BNJ9	155	155	-125.37	0.19	155	150	-224.06	0.30	115	20	-127.11	0.15
P19879	295	90	-110.49	0.18	300	90	-202.68	0.29	330	20	-127.91	0.09
F1N1G1	120	85	-119.64	0.20	355	120	-201.77	0.30	255	65	-147.98	0.09
A6QLI0	115	20	-87.09	0.17	235	10	-166.63	0.15	275	125	-111.12	0.14
P46446	325	15	-88.50	0.16	330	20	-176.13	0.27	130	130	-132.07	0.00
Q3B7M9	195	170	-121.02	0.13	185	175	-208.20	0.21	190	55	-143.45	0.17
P49259	310	105	-133.40	0.12	310	105	-269.78	0.22	175	35	-162.70	0.13
Q9XSC9	210	130	-100.15	0.20	90	140	-192.58	0.21	65	10	-214.34	0.14
Q0VCX4	95	65	-98.64	0.10	90	65	-228.30	0.20	280	60	-121.27	0.03
F1N726	330	5	-63.30	0.14	245	115	-154.40	0.17	235	90	-164.20	0.16
E1BNG2	145	50	-103.49	0.15	100	70	-219.62	0.21	295	90	-171.25	0.16
F1MS76	85	85	-119.39	0.12	80	85	-208.68	0.16	345	100	-129.40	0.16
P02584	210	115	-72.32	0.20	35	100	-161.20	0.25	70	130	-154.84	0.16
Q3SYU8	150	155	-103.78	0.10	155	155	-239.32	0.24	155	150	-172.72	0.17
G3N2P6	220	40	-62.49	0.07	355	140	-213.44	0.01	285	115	-117.07	0.09
F1MZI6	20	60	-96.43	0.14	125	60	-177.30	0.35	185	70	-170.43	0.10
G3N0V0	210	35	-104.16	0.18	220	15	-198.26	0.28	280	110	-147.24	0.06
A7MBI8	50	170	-101.90	0.08	60	170	-173.41	0.13	185	145	-145.68	0.03
P25326	20	15	-103.52	0.16	235	55	-191.82	0.21	15	110	-170.40	0.16
Q0PH99	250	15	-89.98	0.17	20	165	-168.06	0.16	100	55	-144.91	0.17
A6QP36	205	100	-84.36	0.20	5	75	-215.93	0.18	355	90	-169.19	0.17
Q2KJC9	355	100	-110.01	0.06	5	90	-215.77	0.22	80	55	-130.53	0.01
Q29RU4	60	80	-94.91	0.05	65	55	-219.70	0.07	80	75	-128.90	0.18
P50397	315	115	-132.40	0.12	310	120	-294.98	0.22	305	15	-159.81	0.11
Q0VD19	120	75	-122.40	0.20	5	100	-233.08	0.29	190	90	-201.29	0.08
F6S1Q0	295	55	-117.77	0.15	310	50	-227.21	0.24	350	5	-147.35	0.17
P21809	100	95	-138.77	0.19	100	95	-215.89	0.35	180	130	-133.59	0.17
F1MS00	70	75	-63.91	0.04	270	75	-161.02	0.01	270	140	-140.53	0.17
Q5E946	195	25	-87.59	0.22	195	30	-158.70	0.31	100	115	-126.86	0.19
Q5E947	110	80	-91.22	0.19	290	70	-160.88	0.29	15	130	-150.39	0.15
F1N0R8	120	135	-120.58	0.21	15	115	-190.09	0.27	80	125	-162.36	0.01
G3MXK8	355	90	-109.49	0.20	55	140	-187.61	0.27	215	60	-158.65	0.06
H9GW43	220	165	-118.62	0.22	0	95	-170.38	0.26	340	90	-208.23	0.12
G3X6Q8	65	45	-95.99	0.18	80	120	-224.87	0.19	295	55	-157.84	0.01
Q3ZBY4	220	55	-124.33	0.18	215	55	-190.18	0.31	105	70	-130.27	0.18

Q0P5J4	195	140	-88.47	0.15	300	50	-252.48	0.04	200	30	-125.28	0.17
F1MBN4	340	90	-144.22	0.15	340	90	-202.12	0.29	70	75	-124.18	0.04
Q56JW4	350	5	-108.23	0.22	330	15	-161.58	0.32	215	155	-153.58	0.18
F1MNN7	10	165	-89.02	0.16	280	105	-176.23	0.04	355	90	-146.63	0.17
P00741	280	105	-85.16	0.21	265	140	-186.43	0.26	145	120	-150.00	0.15
E1B8L8	100	115	-84.93	0.23	215	50	-167.54	0.07	10	95	-171.11	0.12
P05786	190	90	-111.95	0.09	25	30	-238.58	0.10	100	95	-220.18	0.15
Q0VCM5	275	160	-105.51	0.19	320	10	-164.56	0.19	315	50	-200.53	0.09
A6QNZ7	280	65	-72.56	0.08	100	65	-223.97	0.13	10	100	-196.66	0.15
A0JNP2	60	170	-82.90	0.22	225	155	-124.17	0.31	65	50	-148.90	0.00
G3X6G7	180	50	-100.82	0.14	20	125	-206.31	0.27	310	120	-192.20	0.10
G3X8B1	280	40	-80.17	0.21	340	160	-156.19	0.31	290	125	-119.24	0.14
P45478	40	120	-123.31	0.20	40	120	-195.40	0.32	265	65	-120.46	0.17
Q3ZBZ6	245	55	-95.54	0.02	20	110	-172.08	0.05	325	110	-202.82	0.12
Q95M17	10	160	-141.84	0.19	15	155	-273.55	0.23	50	55	-135.83	0.14
E1BLA8	190	90	-124.36	0.08	190	90	-289.01	0.18	335	80	-115.21	0.17
E1BD63	355	105	-88.15	0.21	350	125	-211.34	0.19	170	155	-194.27	0.05
Q3ZCL0	100	45	-77.42	0.13	100	160	-201.82	0.17	305	55	-163.29	0.15
E1BKK9	130	140	-89.71	0.04	300	55	-170.52	0.32	70	30	-114.48	0.08
P00514	230	100	-127.88	0.04	235	105	-291.54	0.09	100	40	-182.92	0.05
Q3SZZ9	95	115	-63.21	0.10	195	135	-168.83	0.01	320	35	-107.35	0.03
P18892	320	50	-79.16	0.18	170	60	-239.60	0.23	190	25	-111.61	0.15
Q148H6	315	35	-58.70	0.07	310	35	-126.00	0.03	335	60	-147.39	0.15
Q148H7	100	45	-97.03	0.16	100	40	-257.79	0.16	30	110	-141.74	0.09
F1N7D7	110	70	-126.14	0.05	255	45	-321.92	0.18	100	35	-157.23	0.14
Q3SWW8	100	35	-99.18	0.13	100	25	-193.44	0.12	110	70	-181.98	0.02
F1MI18	165	20	-98.76	0.04	240	75	-204.16	0.02	95	115	-135.26	0.07
P01035	190	25	-70.43	0.18	220	45	-133.93	0.26	230	95	-168.12	0.11
A5PJN2	335	60	-85.60	0.18	55	45	-211.08	0.22	300	55	-146.03	0.14
F1MSA6	65	70	-77.65	0.14	45	55	-164.16	0.09	170	60	-154.66	0.10
F1MS32	170	130	-84.90	0.21	260	60	-159.07	0.29	355	130	-139.42	0.15
F1MUT3	330	105	-127.55	0.14	325	110	-253.85	0.24	190	90	-208.63	0.07
F1MKS5	220	40	-72.79	0.02	225	45	-133.08	0.11	10	160	-204.17	0.16
Q0VCU3	305	55	-108.28	0.16	50	160	-205.13	0.19	35	160	-123.73	0.20
P31096	70	30	-73.85	0.10	60	25	-150.66	0.11	45	120	-153.71	0.14
A1L528	280	160	-74.67	0.21	300	60	-166.94	0.21	245	60	-139.75	0.10
Q0P569	165	155	-120.65	0.02	180	80	-243.68	0.06	255	55	-142.23	0.14
P02070	45	60	-84.93	0.19	45	75	-162.71	0.25	10	160	-215.21	0.11
Q9TU03	305	105	-99.15	0.19	150	70	-177.13	0.27	55	95	-178.14	0.14
F1MK08	175	110	-98.98	0.20	135	120	-233.84	0.20	340	95	-125.77	0.07
P42916	330	55	-108.12	0.12	330	60	-283.27	0.20	240	115	-182.72	0.08
Q29RQ1	55	90	-75.65	0.02	40	75	-150.80	0.08	60	85	-153.32	0.17
Q08DU4	5	160	-105.63	0.14	355	160	-173.87	0.26	130	80	-152.02	0.08
F1MM32	170	95	-115.18	0.09	170	95	-214.42	0.19	220	100	-170.55	0.06
P19803	175	65	-80.99	0.15	40	100	-180.26	0.19	175	25	-161.31	0.17
F1MJ00	305	70	-117.97	0.03	310	70	-225.38	0.22	170	95	-176.06	0.09

**E1BAT3**	65	20	-66.43	0.15	280	110	-116.50	0.08	40	105	-134.93	0.16
**F1MZJ5**	280	110	-147.88	0.15	280	110	-246.12	0.28	5	160	-151.77	0.13
**O77834**	65	120	-87.99	0.14	130	75	-181.93	0.28	280	110	-190.78	0.13
**F1MWX8**	60	85	-96.34	0.21	65	80	-225.74	0.28	305	70	-181.32	0.02
**E1B9H5**	240	115	-114.45	0.08	245	115	-277.00	0.09	190	135	-102.97	0.17
**A6QPP7**	45	130	-109.83	0.21	275	35	-197.82	0.28	45	60	-137.57	0.17
**E1BKZ1**	220	100	-117.37	0.09	80	50	-193.72	0.21	330	60	-170.57	0.09
**Q2KJJ7**	55	95	-129.55	0.16	315	125	-211.23	0.28	45	75	-152.76	0.05
**Q2TBL6**	5	160	-136.36	0.14	25	165	-295.69	0.21	150	75	-153.37	0.16
**Q08DN1**	335	90	-82.22	0.16	340	95	-187.37	0.13	175	110	-152.39	0.17
**Q3ZC35**	50	60	-46.90	0.02	155	25	-134.57	0.02	30	60	-116.14	0.17
**Q0P5D6**	220	70	-107.98	0.20	160	165	-211.05	0.26	330	130	-163.23	0.09
**Q2KJI3**	270	80	-92.91	0.12	125	85	-191.32	0.11	345	95	-157.82	0.05
**P55859**	10	155	-94.07	0.20	30	145	-219.13	0.27	325	105	-192.63	0.11
**Q3SZA6**	5	65	-78.84	0.17	10	70	-196.14	0.24	335	45	-136.20	0.14
**A6QP79**	0	0	-0.00	2.00	0	0	-0.00	2.00	270	95	-152.15	0.13
**F1N6D4**	355	110	-91.30	0.19	5	110	-162.57	0.33	175	85	-139.68	0.13
**P62833**	165	70	-82.33	0.19	170	70	-166.24	0.31	255	50	-173.21	0.05
**G3N2K4**	300	95	-122.97	0.15	40	105	-225.45	0.27	115	60	-114.07	0.12
**F1N152**	30	25	-108.16	0.18	40	25	-227.74	0.24	290	135	-160.16	0.07
**F1N3A1**	20	25	-113.25	0.11	25	10	-250.32	0.20	80	105	-117.75	0.17
**P17690**	165	60	-108.02	0.06	165	60	-191.08	0.18	230	155	-174.16	0.10
**G5E5C9**	135	85	-88.32	0.18	135	90	-160.43	0.23	115	25	-142.72	0.03
**G5E5C8**	45	165	-128.36	0.16	30	165	-287.48	0.23	290	45	-213.03	0.14
**O02739**	170	10	-95.16	0.15	65	55	-241.19	0.23	255	70	-167.90	0.12
**P41361**	200	65	-95.82	0.24	130	115	-220.60	0.25	60	30	-181.86	0.18
**Q3SZH5**	220	125	-107.93	0.09	315	20	-196.43	0.29	50	80	-173.49	0.15
**A4FUA8**	90	95	-69.29	0.17	95	100	-175.31	0.24	210	85	-239.41	0.13
**E1BDR2**	290	45	-153.10	0.16	290	45	-267.09	0.30	215	165	-159.67	0.16
**F1MUP1**	255	70	-108.07	0.12	255	75	-242.10	0.21	180	50	-57.47	0.16
**G3N342**	335	110	-100.02	0.07	80	35	-185.54	0.17	65	55	-169.12	0.05
**A5PKJ8**	180	45	-30.27	0.19	320	50	-69.56	0.04	5	160	-197.17	0.11
**E1BE60**	20	160	-117.78	0.19	220	165	-193.87	0.32	135	90	-134.91	0.06
**Q05927**	60	30	-111.20	0.22	55	25	-219.71	0.24	20	90	-118.34	0.16
**Q2TBT6**	75	65	-109.88	0.17	100	85	-255.30	0.14	135	115	-151.55	0.14
**A6QP30**	210	85	-157.56	0.16	210	75	-292.45	0.19	320	25	-169.97	0.14
**Q863B3**	325	105	-135.71	0.09	225	70	-213.06	0.21	305	95	-177.17	0.15
**F1MHQ8**	335	45	-102.49	0.14	305	95	-187.89	0.18	170	70	-151.33	0.12
**Q6B855**	330	90	-92.31	0.17	290	120	-196.34	0.24	0	0	-0.00	2.00
**Q9GKR3**	340	95	-104.42	0.02	35	65	-244.73	0.05	5	110	-158.90	0.13
**Q0VCX2**	335	130	-108.50	0.02	335	135	-219.04	0.09	155	25	-97.21	0.01
**Q0IIG8**	30	65	-72.38	0.19	35	65	-158.08	0.23	305	55	-154.28	0.06
**A5PJJ1**	45	110	-65.94	0.19	180	70	-136.67	0.04	10	70	-138.87	0.14
**F1N720**	290	135	-100.29	0.04	290	135	-199.52	0.15	270	80	-138.37	0.11
**F1MUY2**	200	135	-94.00	0.07	200	135	-263.14	0.11	180	165	-164.28	0.15
**O46375**	220	100	-72.35	0.21	205	90	-139.29	0.28	280	35	-154.99	0.01

A7E3S8	200	50	-90.42	0.12	255	50	-238.89	0.18	25	20	-184.80	0.09
Q3MI05	55	115	-92.61	0.19	155	160	-204.44	0.19	35	25	-175.98	0.15
Q5E9F5	30	45	-98.60	0.16	85	100	-175.12	0.36	125	140	-145.83	0.18
A5D7A0	295	40	-76.52	0.16	295	40	-186.69	0.25	55	120	-125.97	0.12
F1N1D8	250	100	-139.40	0.16	245	55	-247.85	0.27	10	170	-180.88	0.06
Q3SYR8	115	135	-75.11	0.21	115	130	-117.91	0.22	25	155	-130.09	0.06
P05307	220	45	-96.80	0.09	215	45	-199.73	0.19	275	95	-123.44	0.15
G3N0X3	350	80	-125.77	0.14	345	85	-214.10	0.11	10	105	-134.14	0.07
A5PK69	35	105	-119.85	0.16	35	105	-184.43	0.32	75	25	-160.91	0.12
F1MWX3	140	95	-115.95	0.20	125	105	-238.29	0.26	260	30	-171.40	0.12
A6H713	75	95	-117.81	0.22	80	85	-200.41	0.18	135	40	-132.14	0.18
P34933	210	90	-100.36	0.04	195	90	-214.57	0.13	100	30	-146.16	0.16
E1BJS1	55	110	-134.34	0.20	60	110	-219.82	0.31	195	160	-127.75	0.16
G5E604	235	35	-82.83	0.22	190	165	-191.87	0.28	230	125	-144.36	0.09
G3N081	55	35	-83.92	0.21	40	35	-188.12	0.06	70	35	-155.22	0.16
Q3SZ45	355	140	-81.30	0.20	205	55	-182.84	0.27	310	120	-176.73	0.04
A5D7E8	65	35	-100.80	0.07	35	140	-216.78	0.15	35	105	-175.93	0.14
A2I7N0	115	120	-89.00	0.18	110	120	-195.96	0.23	350	85	-194.71	0.13
F1MWU9	355	145	-102.48	0.01	345	25	-232.57	0.23	195	90	-177.29	0.04
Q2HJD5	50	90	-84.52	0.16	20	155	-187.30	0.21	55	110	-173.18	0.18
G3N188	275	100	-94.73	0.11	235	40	-149.21	0.25	75	90	-181.11	0.16
P05689	5	170	-96.05	0.08	25	170	-248.29	0.22	125	105	-159.07	0.14
G3X6N3	115	120	-105.50	0.18	130	70	-201.24	0.17	115	135	-103.65	0.18
Q1JP75	30	65	-82.39	0.16	125	60	-163.01	0.25	250	95	-217.87	0.14
Q28034	110	55	-97.81	0.04	80	20	-212.20	0.10	85	100	-168.32	0.17
A6QPT4	180	65	-124.54	0.19	260	30	-246.68	0.20	295	40	-141.95	0.11
Q2KJ53	180	145	-77.15	0.20	15	105	-177.09	0.28	220	45	-160.42	0.09
E1BLR9	170	95	-53.57	0.02	175	95	-137.59	0.09	220	95	-155.23	0.06
Q2KIG3	50	75	-115.94	0.20	210	70	-228.06	0.26	180	50	-169.12	0.12
E1BNK3	90	60	-107.79	0.16	245	70	-178.26	0.16	170	150	-150.81	0.02
Q3MHX0	85	165	-102.97	0.17	45	115	-208.31	0.17	75	115	-158.79	0.07
F1MX87	55	30	-100.26	0.10	60	35	-224.83	0.20	30	85	-113.83	0.17
F1MLQ1	195	130	-93.63	0.05	190	125	-191.43	0.09	200	130	-119.59	0.01
O62829	80	155	-133.26	0.15	65	140	-224.52	0.24	275	105	-112.94	0.00
A6QL75	280	145	-108.22	0.08	210	110	-206.91	0.23	325	60	-176.57	0.11
F1N0F2	285	155	-93.61	0.18	285	150	-202.83	0.21	290	140	-195.19	0.13
P61585	135	60	-79.35	0.18	140	55	-168.10	0.25	60	45	-236.07	0.09
P22226	275	30	-115.20	0.20	275	30	-167.32	0.37	290	120	-150.36	0.14
Q8MJ24	230	50	-143.42	0.13	230	50	-253.10	0.28	120	30	-165.74	0.17
Q05717	275	140	-137.72	0.20	275	135	-220.84	0.27	335	120	-240.28	0.08
Q3T0L5	290	160	-86.90	0.20	135	80	-165.48	0.24	85	135	-128.02	0.16
P21214	150	55	-108.28	0.16	25	45	-287.11	0.20	20	120	-145.83	0.09
F1MW79	240	100	-79.52	0.11	245	120	-208.83	0.24	275	140	-192.71	0.17
P02662	65	40	-140.87	0.20	60	40	-277.69	0.30	130	45	-113.49	0.16
P02663	330	60	-112.16	0.06	350	55	-231.05	0.12	230	50	-247.29	0.13
F1MJ28	165	100	-122.23	0.05	165	100	-269.98	0.16	275	30	-152.87	0.16

**P12799**	95	100	-61.17	0.14	200	130	-205.09	0.03	145	105	-118.97	0.01
**Q28085**	150	100	-64.92	0.06	275	105	-130.09	0.07	75	125	-138.85	0.17
**P55052**	50	115	-63.88	0.21	30	85	-153.83	0.32	245	120	-154.66	0.12
**G3MXM5**	70	115	-112.74	0.13	75	115	-185.01	0.27	25	45	-176.50	0.07
**Q3SYT3**	65	145	-112.06	0.00	25	115	-186.69	0.15	90	60	-167.75	0.15
**E1BF81**	55	95	-74.79	0.15	110	135	-201.97	0.22	205	70	-175.24	0.03
**Q07537**	125	30	-127.86	0.18	140	30	-202.84	0.26	175	95	-89.45	0.06
**E1BBY7**	335	120	-150.27	0.07	335	120	-263.45	0.20	80	155	-176.07	0.15
**G5E5P3**	285	120	-90.10	0.16	290	120	-193.80	0.28	280	145	-151.17	0.06
**E1BBP4**	310	110	-104.08	0.18	180	50	-219.98	0.25	85	50	-164.19	0.15
**Q27965**	220	95	-107.52	0.01	55	30	-198.01	0.19	190	130	-152.83	0.09
**F1ML13**	305	120	-110.46	0.08	25	130	-185.85	0.13	180	160	-150.73	0.13
**P80457**	135	105	-131.47	0.10	325	110	-256.47	0.22	355	50	-114.46	0.20
**F1N2J8**	30	95	-116.27	0.17	30	90	-214.07	0.19	335	110	-214.71	0.10
**F1N2J9**	335	80	-71.62	0.19	335	85	-151.24	0.29	140	155	-218.16	0.02
**Q2HJH2**	205	120	-95.89	0.19	295	60	-155.47	0.21	165	110	-234.52	0.02
**A6QQQ9**	190	90	-95.80	0.13	185	90	-228.67	0.20	40	60	-178.27	0.07
**F1N1Z8**	240	125	-74.01	0.18	180	65	-186.88	0.30	310	50	-147.95	0.06
**F1MMR6**	100	75	-109.16	0.21	110	80	-198.71	0.23	150	75	-213.22	0.15
**E1BFD0**	325	60	-103.48	0.15	330	60	-200.49	0.23	0	0	-0.00	2.00
**A5D7V3**	185	120	-78.12	0.19	45	40	-200.67	0.24	315	20	-147.85	0.10
**Q3T145**	340	145	-74.48	0.21	10	55	-164.19	0.32	210	110	-140.03	0.15
**F1MPE1**	250	85	-109.89	0.06	185	30	-204.82	0.13	185	145	-118.26	0.05
**Q3ZC84**	350	90	-102.05	0.20	340	125	-227.84	0.28	85	70	-122.39	0.03
**Q0IIA2**	105	60	-85.29	0.20	55	135	-177.26	0.28	160	145	-202.76	0.19
**Q3T0P6**	85	135	-103.36	0.18	190	80	-199.84	0.19	185	70	-141.72	0.18
**A7E350**	0	0	-0.00	2.00	0	0	-0.00	2.00	305	115	-126.18	0.18
**A5D7D5**	280	60	-76.36	0.04	310	60	-157.96	0.13	45	40	-110.26	0.16
**G3N3D4**	145	75	-154.40	0.13	160	70	-217.77	0.24	325	65	-151.38	0.15
**F1N5M2**	300	165	-109.93	0.16	35	65	-222.26	0.19	95	75	-144.66	0.16
**A6QLB7**	160	150	-179.78	0.23	60	100	-185.96	0.38	240	135	-128.43	0.17
**F1MFJ3**	115	55	-60.14	0.09	90	70	-157.96	0.06	165	85	-136.68	0.16
**P79345**	185	70	-100.71	0.23	185	70	-153.93	0.37	250	80	-158.61	0.10
**E1BG25**	210	110	-80.45	0.17	60	120	-212.74	0.15	350	90	-174.63	0.16
**F1MZ96**	145	65	-88.93	0.18	80	75	-173.70	0.18	335	80	-123.09	0.16
**A1A4R1**	315	40	-87.98	0.10	165	125	-167.09	0.17	325	110	-189.86	0.08
**A5D7M6**	170	110	-133.61	0.08	165	110	-319.16	0.09	35	95	-168.83	0.08
**E1BDY3**	225	155	-120.44	0.11	35	10	-271.94	0.09	190	90	-211.24	0.11
**F1N1W3**	295	125	-123.80	0.18	335	110	-235.18	0.23	180	60	-128.93	0.17
**P02754**	135	25	-70.09	0.21	160	25	-168.47	0.25	205	120	-140.23	0.15
**A0JND2**	140	110	-71.72	0.11	175	90	-236.04	0.03	140	120	-163.25	0.14
**F1MUX5**	325	50	-100.40	0.20	75	120	-200.54	0.24	345	100	-138.75	0.08
**G3N1H5**	355	150	-129.40	0.19	135	80	-206.35	0.23	230	95	-175.74	0.05
**Q3ZBD7**	260	110	-117.06	0.18	190	105	-240.67	0.22	260	80	-176.35	0.11
**Q5E9I6**	300	35	-82.37	0.23	190	115	-162.74	0.27	180	45	-180.44	0.18
**Q2HJ49**	295	100	-119.05	0.08	295	100	-243.77	0.21	20	125	-139.18	0.06

**Q28115**	175	85	-104.76	0.09	225	150	-200.05	0.23	0	85	-180.67	0.08
**P58073**	25	30	-112.90	0.22	25	30	-187.12	0.33	135	160	-149.02	0.18
**P02668**	195	65	-97.36	0.19	255	95	-152.15	0.32	215	60	-201.82	0.15
**Q2TBU0**	265	30	-77.57	0.07	265	30	-173.39	0.17	330	110	-192.62	0.15
**E1BE76**	30	120	-114.29	0.14	20	130	-216.69	0.27	175	85	-157.61	0.08
**F1MHF1**	250	110	-107.50	0.14	95	115	-199.31	0.25	25	30	-170.86	0.19
**P18915**	295	110	-86.26	0.20	150	125	-202.90	0.33	170	65	-150.77	0.17
**A6QLL8**	220	60	-133.50	0.22	220	60	-189.03	0.38	250	110	-158.92	0.12
**Q2KJ32**	150	40	-114.38	0.21	330	110	-191.47	0.36	30	125	-181.26	0.15
**Q3MHN5**	135	160	-111.77	0.19	35	65	-203.05	0.16	255	95	-140.57	0.11
**G3N0S9**	350	60	-101.30	0.13	230	95	-223.46	0.15	260	35	-121.98	0.10
**A6QLZ7**	235	30	-120.35	0.19	260	80	-225.89	0.27	175	90	-145.28	0.01
**P81265**	65	55	-84.42	0.08	75	55	-173.12	0.16	290	15	-129.55	0.18
**P00727**	135	120	-108.88	0.15	140	120	-237.66	0.27	295	100	-196.15	0.09
**F1MKM4**	65	65	-103.70	0.22	0	85	-189.93	0.21	350	145	-173.06	0.16
**A5D7C8**	340	155	-85.99	0.10	215	85	-243.33	0.11	80	120	-165.97	0.13
**P17248**	180	45	-133.36	0.22	95	90	-197.33	0.23	250	115	-163.43	0.10
**E1BF27**	240	150	-106.30	0.07	230	80	-261.13	0.12	240	55	-150.23	0.16
**A6QM01**	60	70	-124.10	0.14	65	65	-203.06	0.28	305	95	-170.89	0.15
**G3N2D7**	55	105	-80.24	0.20	240	105	-148.23	0.27	350	125	-191.53	0.10
**Q2KJF1**	70	155	-129.14	0.05	85	145	-179.99	0.06	295	155	-123.12	0.16
**Q29443**	115	130	-103.92	0.16	60	120	-195.57	0.28	290	15	-211.06	0.11
**E1BMJ0**	210	115	-84.56	0.14	60	115	-158.81	0.24	230	155	-131.47	0.17
**A5PKH0**	205	80	-76.99	0.20	25	50	-151.42	0.10	355	170	-186.02	0.15
**Q95LA9**	310	140	-103.50	0.17	25	125	-188.36	0.21	260	140	-155.05	0.05
**F1MBF6**	295	70	-88.04	0.08	20	55	-163.07	0.30	265	150	-130.89	0.02
**Q08D91**	195	130	-111.34	0.03	210	150	-308.08	0.07	50	135	-168.36	0.10
**F1N7T2**	175	110	-114.79	0.20	175	115	-213.58	0.26	125	55	-127.14	0.17
**Q3SYW7**	50	140	-71.27	0.13	40	140	-172.31	0.14	180	130	-136.27	0.17
**Q148J4**	180	130	-70.38	0.20	175	130	-173.63	0.29	45	15	-131.58	0.18
**F6QLM5**	120	65	-74.20	0.22	20	155	-209.33	0.27	300	65	-124.36	0.07
**A4IFP7**	270	50	-97.24	0.22	155	115	-202.89	0.28	230	150	-154.38	0.04
**P80109**	180	145	-129.19	0.15	285	15	-281.27	0.22	40	80	-108.05	0.09
**Q5E956**	225	155	-90.03	0.17	235	155	-172.71	0.33	115	85	-155.26	0.10
**A5PJE3**	350	125	-96.37	0.13	345	130	-228.77	0.14	175	110	-175.45	0.18
**G5E5H2**	295	155	-77.94	0.19	90	105	-184.29	0.26	215	150	-185.87	0.11
**A6H797**	195	60	-74.26	0.19	305	130	-180.74	0.23	65	65	-169.37	0.15
**Q5E9G3**	340	130	-107.74	0.03	300	100	-227.21	0.14	230	80	-173.96	0.05
**P13384**	265	150	-101.22	0.04	260	150	-167.91	0.06	325	20	-131.73	0.14
**F1N6Y1**	50	135	-109.05	0.09	205	90	-217.69	0.17	75	155	-147.73	0.11
**F1MLR4**	260	140	-93.27	0.01	290	130	-180.96	0.02	60	120	-162.30	0.14
**E1BIT9**	240	55	-105.14	0.22	345	170	-319.30	0.21	220	25	-126.61	0.18
